# Radiolabeled nanomaterial for cancer diagnostics and therapeutics: principles and concepts

**DOI:** 10.1186/s12645-023-00165-y

**Published:** 2023-02-27

**Authors:** Muskan Goel, Yuri Mackeyev, Sunil Krishnan

**Affiliations:** 1 Amity School of Applied Sciences, Amity University, Gurugram, Haryana 122413 India; 2 Vivian L. Smith Department of Neurosurgery, University of Texas Health Science Center, Houston, TX 77030 USA

**Keywords:** Nanotechnology, Radiolabeled nanomaterial, Nano-radiopharmaceuticals, Radionuclides, Cancer

## Abstract

In the last three decades, radiopharmaceuticals have proven their effectiveness for cancer diagnosis and therapy. In parallel, the advances in nanotechnology have fueled a plethora of applications in biology and medicine. A convergence of these disciplines has emerged more recently with the advent of nanotechnology-aided radiopharmaceuticals. Capitalizing on the unique physical and functional properties of nanoparticles, radiolabeled nanomaterials or nano-radiopharmaceuticals have the potential to enhance imaging and therapy of human diseases. This article provides an overview of various radionuclides used in diagnostic, therapeutic, and theranostic applications, radionuclide production through different techniques, conventional radionuclide delivery systems, and advancements in the delivery systems for nanomaterials. The review also provides insights into fundamental concepts necessary to improve currently available radionuclide agents and formulate new nano-radiopharmaceuticals.

## Introduction

Nuclear medicine, a field that utilizes radionuclides emitting particulate radiation (α, ß, proton, neutron, etc.) and/or magnetic waves (X-rays, γ rays), has revolutionized the diagnosis and treatment of cancer (Farzin et al. 2018). Radionuclides such as α, ß-, and Auger electron emitters, when localized within tumors, can serve as therapeutic agents by causing targeted destruction and elimination of exposed cancer cells (Luderer et al. 2015). On the other hand, single photon (gamma ray) emitters and positron emitters, when localized within tumors, can serve as diagnostic agents when the emitted gamma ray or annihilation gamma ray pairs are sensed by detector arrays positioned around the patient (Fass 2008). Often, a radionuclide pair allows for both diagnosis and therapy with one isotope serving as the imaging agent and the other one serving as the therapeutic agent. This versatility in imaging and treating cancer(s) has made nuclear medicine a hot area of research with constant development and validation of newer agents. A host of these radionuclide imaging and/or therapy agents have been cleared by the FDA and are in widespread clinical use across a spectrum of indications.

While radionuclides can readily be viewed as the ultimate nanoparticle where the working unit, the radioactive element, is already scaled down to the atomic level; a new discipline of science has evolved in parallel in the last two decades based on the recognition that naturally occurring or synthetic materials that are tiny fragments of the same bulk materials possess unique physical, chemical, and functional properties by virtue of their small size. These nanomaterials, defined loosely as ones measuring less than 100–1000 nm in one dimension, have large surface area to volume ratios compared to bulk materials. In turn, this attribute creates a higher probability, per unit mass, of interaction of surface atoms with atoms in adjacent substances or with perturbations caused by incident energy. In a classic example, stained glass windows have hues of different colors when the glass is admixed with different nanoscale elements; these colors imparted by the nanoscale elements are distinctly different from that of their bulk elements due to distinct interactions with visible light by the surface atoms of the nanomaterial. Extending this observation to more recent applications, just changing the size of semiconducting quantum dots results in clearly noticeable changes in their fluorescence color or emission wavelength. Bringing this exciting new nanotechnological frontier to the realm of biological applications has spawned a new era of nanomedicine that promises to revolutionize how we diagnose and treat diseases in the clinic (Pelaz et al. 2017). The nanoparticles studied and deployed in biological context have included synthetic lipid nanoparticles (liposomes, micelles, uni- and multi-lamellar vesicles, exosomes, etc.), highly ordered polymers, dendrimers, metals of various geometries, core–shell constructs, multilayer and/or multifaceted particles, and such. A handful of these constructs designed to ferry therapeutic payloads to diseased tissues in the body have cleared the FDA and shown improved clinical efficacy and/or tolerability compared to free agent alone.

Importantly, however, this renaissance in nanotechnology applications in biology and medicine has transpired quietly and organically in parallel with advancements in nuclear medicine with minimal, if any, convergence or overlap. To some extent, this is because both fields were nascent but burgeoning, content will be looking inward rather than outward and very focused on their own agendas and aspirations. As both fields have matured, however, there is a unique opportunity to look at potential overlap, cross-fertilization, and synergies. As such, there are increasing forays from one discipline to the other and attempts to acquire skills from the other and/or assimilate the lessons learned from the other that foretell the emergence of a new class of agents that we refer to as ‘nano-radiopharmaceuticals’ (Nie et al. 2007; Farokhzad and Langer 2009; Prasad 2012; Chen et al. 2016a).

This integrated discipline that coalesces radionuclides and nanomaterials forebode the emergence of a new array of nano-radiotracers for imaging and diagnostics, nano-radiopharmaceuticals for therapy, and nano-delivery constructs that overcome barriers to delivery of radionuclides to their desired destinations. Given how new and fresh the field is, it is not surprising that most of the work to date has been in the realm of preclinical research (Kunjachan et al. 2015) with little that has advanced to clinical adoption (Min et al. 2015).

An added attribute of radiopharmaceuticals is their ability to be used for both imaging and therapy, termed as ‘theranostics’ (Lim et al. 2015). In turn, this capability serves as a step towards the development of personalized medications that can monitor and treat a disease simultaneously (Mura and Couvreur 2012; Chen et al. 2017a; Jo et al. 2016). In some instances, it may be preferable to decouple the diagnostic and therapeutic functionalities of a radiopharmaceutical to minimize unnecessary radiation exposure. Herein, radiopharmaceuticals that possess only one mode of decay may be more suitable for just diagnosis or just therapy. In scenarios where after treatment for a given cancer there is residual disease that needs to be both imaged and treated, a theranostic radionuclide pair may be especially advantageous. As a corollary, nanotheranostics combines nuclear medicine and nanoscience to diagnose and treat cancer simultaneously using nanoformulations of radionuclides (Hamoudeh et al. 2008).

There are several review articles highlighting the development of nanomaterial-based radionuclide agents for imaging (Lee et al. 2013a), therapy (Song et al. 2017), and delivery (Hamoudeh et al. 2008; Smith and Gambhir 2017; Mitra et al. 2006; Polyak and Ross 2018; Peltek et al. 2019). However, these articles highlight specific aspects of nanoradiopharmaceuticals and provide selective updates on the underlying basic principles. A deeper understanding of generalized principles can provide fundamental insights into developing nano-radiopharmaceuticals, compare and contrast agents used currently, identify gaps in knowledge, highlight opportunities to solve these problems, and advance paradigms for personalized medicine modeled on optimally formulated diagnostic, therapeutic, and/or theranostic agents.

This review provides a broad overview of the spectrum of clinically relevant radionuclides, their production methods, conventional radionuclide delivery systems, basic principles of nanomaterial use in biomedical applications, and the promise of utilizing nanomaterials for the advancement of radiopharmaceuticals. The article then highlights various basic concepts encompassing nano-radiopharmaceuticals, reviews methods of radiolabeling nanomaterials, and provides a descriptive synopsis of the applications of radiolabeled nanomaterials.

## Types of radionuclides

**Fig. 1:**
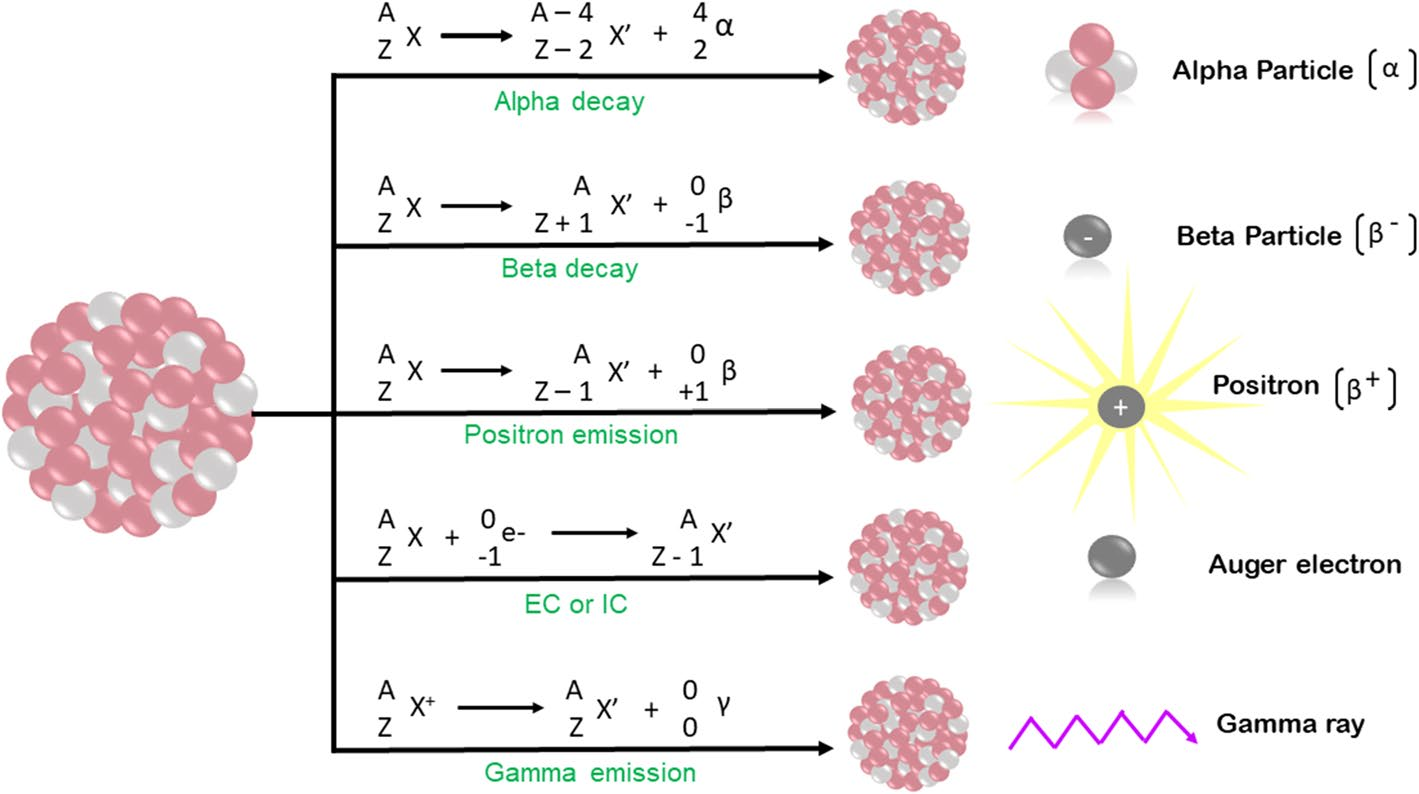
Schematic representation of the underlying nuclear decay that results in production of α, ß-, ß + , Auger electron, and γ radioactive emissions. EC = electron capture, IC = internal conversion

Radioactivity refers to subatomic particles and electromagnetic rays arising from an unstable atomic nucleus. Radioactive decay is the conversion of an unstable nuclide (parents nuclide) to a stable daughter nuclide (if the daughter nuclide is unstable, it further decays). Radioactive decay produces radiation which is a combination of particles and electromagnetic rays. Radioactivity leads to α, ß −, Auger electron, ß + , and γ radioactive emissions (Kassis and Adelstein 2005; Kassis 2008). The underlying nuclear emission leading to the production of different radionuclides is depicted in Fig. 1.

Radionuclides can be classified based on their use in diagnostics, therapeutics, or theranostics. The potential biomedical uses of radionuclides are governed by their physical and biochemical characteristics. The physical features include effective half-life, decay mode, and emission properties (Volkert et al. 1991), while biochemical characteristics include in-vivo stability, toxicity, etc. Apart from these physical and biochemical characteristics of radionuclides, the targeted tumor type, radionuclide affinity, heterogeneity, vascularity, and other clinical and physiological factors also play an essential role in the form of clinical application of a radionuclide. From a utilitarian standpoint, logistical considerations such as local availability, cost, ease of production, purification, storage, transport, good manufacturing practice fabrication, regulations, and nuclear waste disposal also dictate how a given radionuclide is employed clinically (Zimmermann 2013). Lastly, the experience of the practitioner, the relative abundance of ongoing clinical and preclinical investigations, and the prevalence of a given cancer studied at the institute also drive adoption of radionuclides in routine clinical practice.

### Radionuclides for SPECT and PET-based diagnostics

**Fig. 2:**
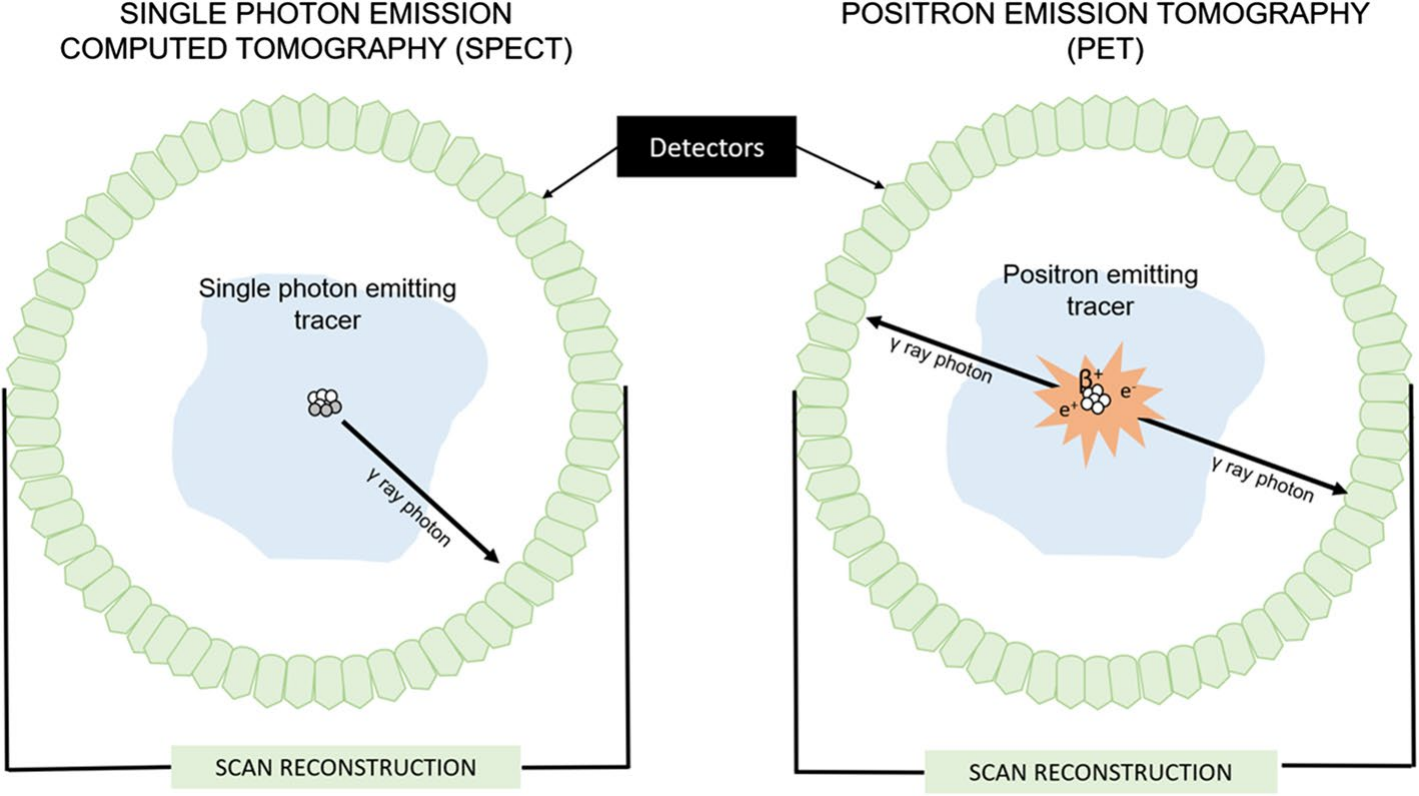
Schematic representation of SPECT (left) and PET (right) imaging

Diagnostic imaging of cancer using radionuclides is conducted with the help of single photon emission computed tomography (SPECT) or positron emission tomography (PET), in which γ rays or ß + emitting radionuclides are employed as depicted in Fig. 2. For SPECT-based imaging, the radionuclide should emit γ rays with 100% abundance (emitting one γ-ray per decay) and an energy range between 100 and 370 keV with negligible particulate radiations emission or higher energy γ-rays (Ting et al. 2010). Ideal radionuclides for SPECT imaging have half-lives that allow for imaging within a few hours after administration and stay concentrated within the tumor of interest for the duration of imaging. For PET-based imaging, positron (ß +) emitting radionuclides are employed which ideally emits low-energy, short-range ß + with a 100% abundance and no higher energy prompts. These positrons instantly combine with an electron, the resulting electron–positron annihilation generates two γ ray photons that travel in diametrically opposite directions with identical energies of nearly 511 keV (Velikyan 2018; Kuntić et al. 2016; Mattoli et al. 2021; Garg et al. 2021; Chen et al. 2021) that are picked up by a circular array of detectors placed around the patient.

### Radionuclides for therapeutics

For therapeutic purposes, radionuclides that emit α, ß-, or Auger electrons are employed. The ideal radionuclide used for these scenarios is one with an effective half-life (the interaction of physical and biological half-lives) longer than the time required for its preparation, introduction, and localization to the tumor site. The range of effective half-lives is usually between 7 h and 7 days (Qaim 2001). Besides the effective half-life, other characteristics like variable energy, penetrating range, and linear energy transfer of particulate radionuclides also play a significant role in utilizing a given radionuclide for therapeutic purposes (Poty et al. 2018). Logistical considerations are similar to those noted above for imaging radionuclides.

### Radionuclides for Theranostics

For theranostics, radionuclides should have characteristics for therapeutic and diagnostic purposes. The ideal radionuclide used in these scenarios is one that generates α, ß-, or Auger electrons for treatment and ß+, or γ radiations for imaging, with the γ radiation optimally around 140 keV so that they can be detected by cameras with minimal background (Nolte et al. 2020). Accordingly, this is accomplished using a single radionuclide or a combination of radionuclides or a theranostic pair where the diagnostic agent is radiolabeled with an imaging radionuclide and the therapeutic agent is radiolabeled with a physiochemically matched radionuclide.

### Production of radionuclides

Traditional methods of radionuclide production were costly and time-consuming. However, with advancements in technology, the production process has been eased considerably. Current methods rely on the use of cyclotrons, nuclear fission, neutron activation, or generators. These are outlined in greater detail in the sections below.

#### Cyclotron

**Fig. 3:**
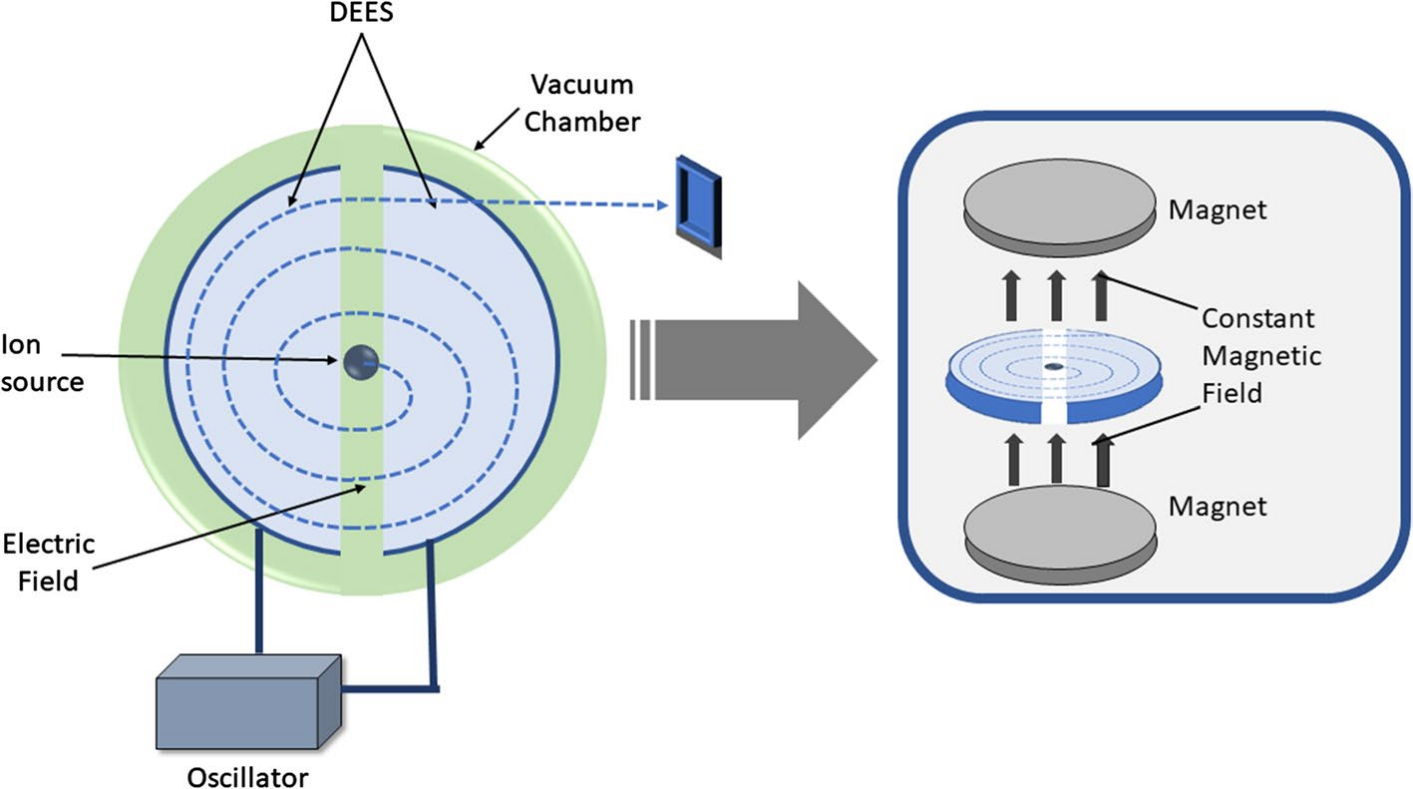
Schematic representation of a cyclotron

A cyclotron is a particle accelerator as depicted in Fig. 3. A high voltage is targeted on an ion source to produce particles that are then accelerated at a high speed in a spiral trajectory until they are extracted and directed towards a target (Peach et al. 2011). Cyclotrons are significant producers of positron-emitting radionuclides either lacking neutrons (protons, deuterons, tritons) or decay by electron capture (EC) (Currie et al. 2011).

#### Nuclear Fission

**Fig. 4:**
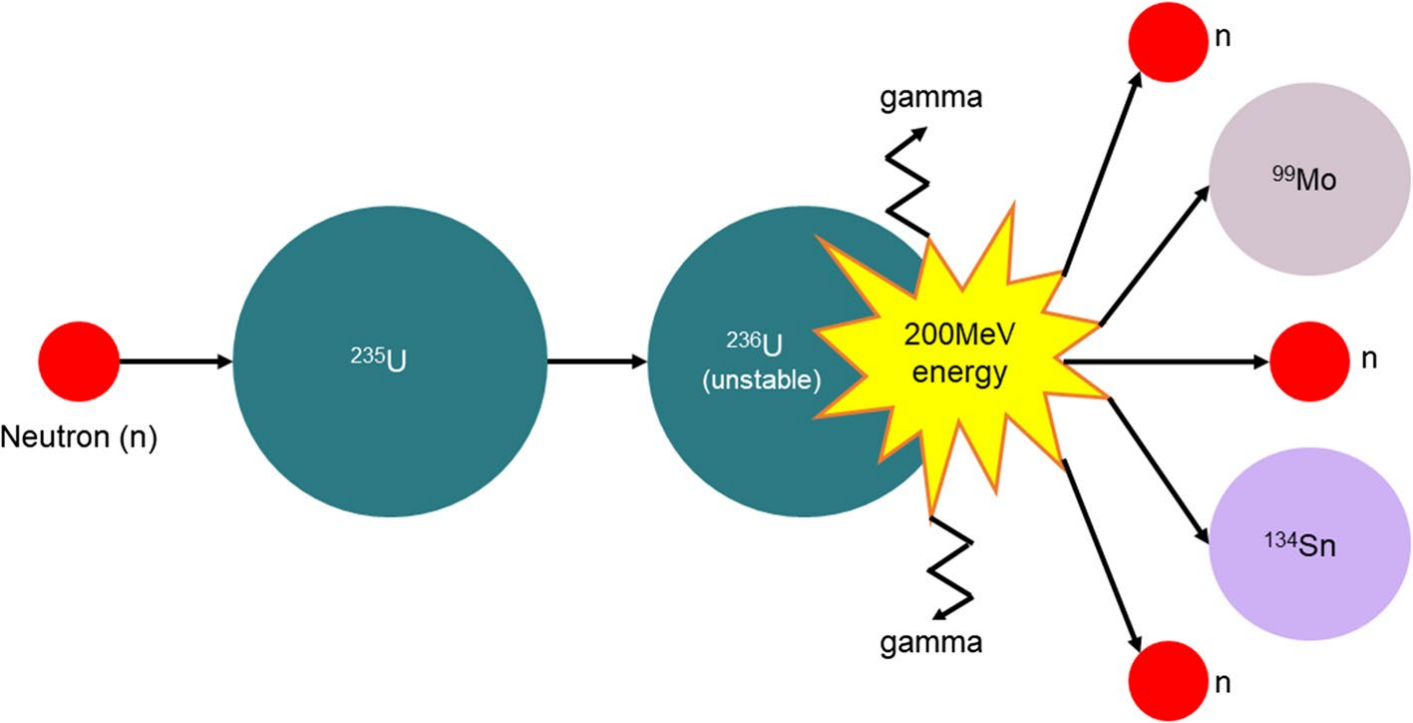
Schematic representation of a fission reaction

In nuclear fission, a neutron is bombarded on a stable target nuclide which generates a highly unstable nuclide as depicted in Fig. 4. This metastable nuclide in the next step produces a pair of atoms, 2–3 neutrons, and γ-ray emissions after undergoing nuclear fission (Currie et al. 2011; Willowson 2019).

#### Neutron activation

**Fig. 5:**
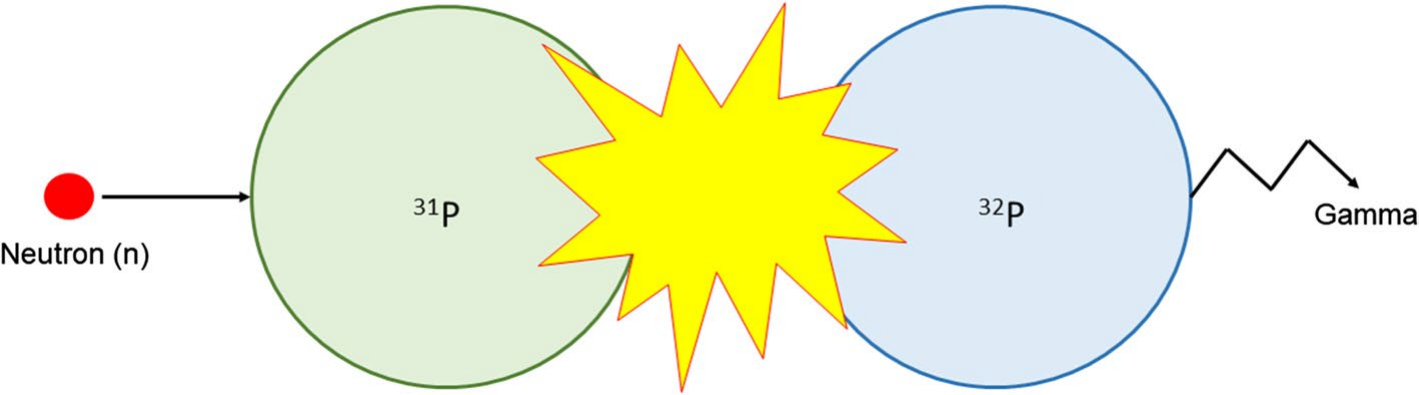
Schematic representation of neutron activation

Like nuclear fission, this process is also conducted in a nuclear reactor and involves the bombardment of neutrons on a stable target nuclide as depicted in Fig. 5. The excited target then returns to the ground state by emitting γ photons and simultaneously producing a radioactive isotope of the same element [(n,γ) reaction]. Alternatively, (n,p) reactions can also be carried out. However, instead of an isotope of the same element resulting from the interaction, the starting target and the end product are different elements (Currie et al. 2011).

#### Generator

**Fig. 6:**
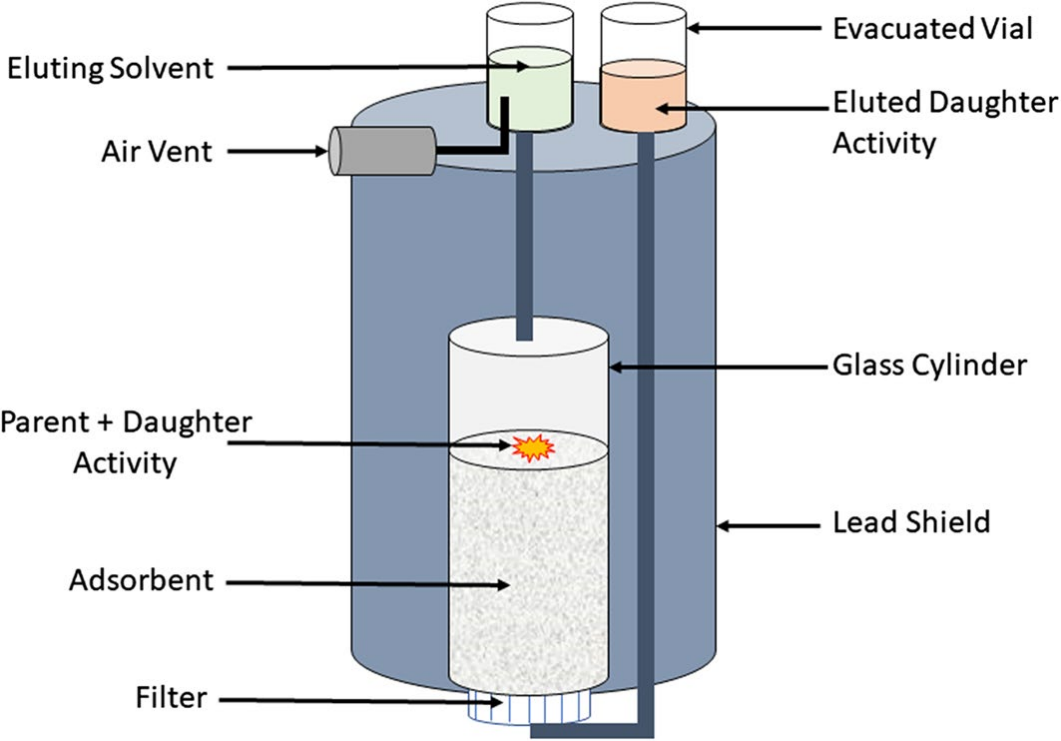
Schematic representation of a generator

A generator contains a solid matrix for the adsorption of a pair of radionuclides as depicted in Fig. 6. A solvent elution method used here aids in selective extraction of daughter nuclides from the matrix. The separation of the two adsorbed radionuclides is based on the physical and chemical properties of these radionuclides. The significant advantages of this equipment are its small size, simple setup, and cost-efficiency (Currie et al. 2011; Dash and Chakravarty 2019). Moreover, it helps produce short half-life radionuclides 'on-site,' e.g., ^62^Cu, ^82^Rb. However, only a few radionuclides can be produced using this technique.

## Basic concepts for nano-radiopharmaceuticals

### Conventional radionuclide delivery

For more than twenty years, radionuclides have been researched and investigated for the treatment and diagnosis of cancer. When employed for cancer treatment, this is called radionuclide therapy or targeted radionuclide therapy and the agents are often termed radiopharmaceuticals.

Conventionally, the radionuclide delivery agent consists of a radionuclide bound to a specific vector with or without using a chelator. Here, the vector binds to the target site (specific epitopes on cancer cells, specific affinities of cancers to certain heavy metallic elements, etc.). The major vectors studied include peptides, protein scaffolds (Boersma and Plückthun 2011; Löfblom et al. 2010), monoclonal antibodies, antibody fragments, and aptamers, (Lipi et al. 2016). Interestingly, the use of an antibody as a vector for radionuclide therapy has been so extensively studied, that the field is specifically termed as radio-immunotherapy (Jabbour et al. 2015; Tsai and Wu 2018; Dash et al. 2015). Similarly, extensive use of peptides as carriers has led to development of a field called Peptide Receptor-based Radionuclide Therapy (PRRT).

However, the use of conventional vectors leads to certain limitations. For example, antibodies may have limited penetration, slow pharmacokinetics, radionuclide decay before radiopharmaceutical localization, non-specific normal organ localization, and high background activity. Similarly, peptides may have fast blood clearance, high tumor-to-background ratio, and non-specific affinity towards normal tissues. These limitations can potentially be overcome when molecular radionuclides are combined with pre-targeted nanomaterials, as discussed below in Section “Methods of radiolabeling nanomaterials” (Stéen et al. 2020).

#### Dosing radionuclides

A key consideration in therapeutic treatments or diagnostic imaging is the issue of dose required or desired for clinical use. Two metrics are typically used in defining the dose used in a clinical setting—the total dose of radioactivity delivered and the time required to deliver this dose. The latter depends on the radionuclide's specific activity, which is measured per gram of compound or the radionuclide's molar activity, which is measured per mole of compound (Coenen et al. 2018). A high specific/molar activity suggests that a lower amount of radioactive compound is required to achieve the same radiation dose delivered. In other words, increased radionuclide activity is achieved at lower concentrations (Wagner and Langer 2011). For formulations with high specific and molar activity, the effectiveness of treatment may possibly be greater in some instances but greater accuracy is needed while measuring radionuclide delay since small errors can propagate to large inaccuracies in delivered dose and lack of tumor specificity could result in unintentionally high doses of radiation delivered to normal tissues and organs. Therefore, this is a metric that has important clinical implications and could explain variability in results obtained by different teams administering the same radionuclide and the same total dose to a given tumor.

#### Self-dose, crossfire effect, non-uniform dose, bystander effect of radionuclides

**Fig. 7:**
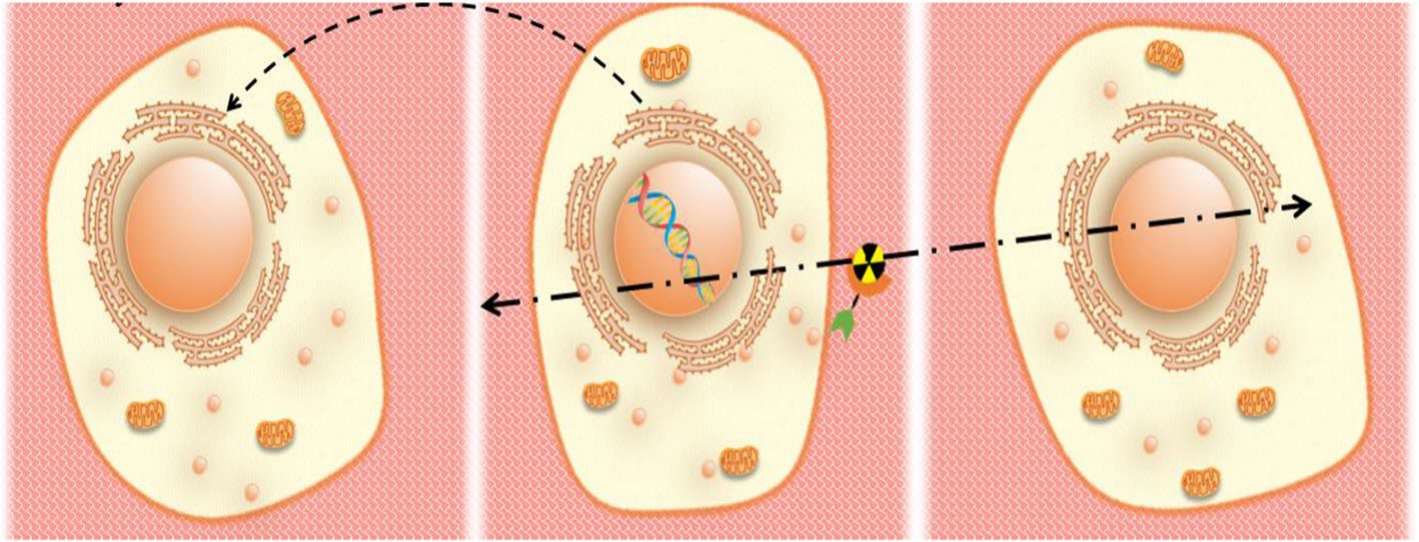
Schematic representation of the bystander (left panel), self-dose (middle panel), and crossfire effect (right panel) effects of radionuclide therapy

In radionuclide therapeutics, the radionuclide emits radiations which are then absorbed by the targeted tissue. This then leads to a biological effect observed within the tissue, be it a molecular lesion (typically DNA damage), clonogenic survival (preservation of the ability of a cancer cell to divide and form a viable colony of cells), delay in mitotic division, programmed cell death, or senescence. (Kassis and Adelstein 2005; Kassis 2008). Self-dose, crossfire effect, non-uniform dose, and bystander effect of radionuclides are events that occur at the tissue level as depicted in Fig. 7.

When radionuclides directly irradiate the targeted cells, this is called self-dose as depicted in Fig. 7 middle panel. Due to the different linear path lengths of α, β, and Auger electrons, self-dose may be dependent on the position of the radionuclide on the tumor cell (for α, β particles) or on the distance between the position of the radionuclide and the DNA (for Auger electrons). This is because Auger electrons do not often travel very far and they need to interact with DNA to produce an effect. The crossfire effect occurs when cells are irritated by the radionuclides present on neighboring or distant cells as depicted in Fig. 7 right panel. As the targeted tissue non-uniformly absorbs radionuclides, tissues have a non-uniform dose (Kassis and Adelstein 2005; Kassis 2008). Cells that are not directly irradiated but are affected by the neighboring irradiated cells experience the bystander effect as depicted in Fig. 7 left panel (Marín et al. 2014).

### Nanomaterials as carriers for radionuclides

Nanomaterials have generated much enthusiasm in the field of science and medicine (Farokhzad and Langer 2009; Prasad 2012; Chen et al. 2016a). In early incarnations, nanomaterials were initially used as delivery agents to deliver large doses of medication. Later, nanomaterials were tagged with radionuclides to study pharmacokinetics, pharmacodynamics, and *in-vivo* biodistribution. With time, nanomaterials bound to radionuclides have shown increasing promise in cancer treatment. A compact synopsis of this field is somewhat complicated by many different ways it has been referred to in the literature, ranging from radionuclide-activated nanomaterials/nanomedicine, nano-radiopharmaceuticals, radio-nanomedicine, etc. (Lee et al. 2013a; Song et al. 2017; Smith and Gambhir 2017; Mitra et al. 2006; Polyak and Ross 2018). Radionuclides could be bound to nanomaterials through various methods, which are discussed in the Section “Methods of radiolabeling nanomaterials”.

#### Advantages of radiolabeled nanomaterials

Across these formulations, a unifying theme is that nanomaterials enjoy high surface area-to-volume ratios, high radionuclide loading and labeling efficiency, and facile synthetic routes to generate constructs with tunable physico-chemical properties, shape, and size. Aside from the aforementioned properties, nanomaterials have some additional attributes that make them potentially attractive for clinical utilization. One of these is the ability to multiplex diagnostic and therapeutic radionuclides onto the same nanomaterial backbone to create facile nanotheranostics. Another angle of this versatility in fabrication of nanomaterials is the ability to functionalize the vector with homing moieties that bind to multiple receptors overexpressed on a tumor. This allows the multi-functionalized radiolabeled nanomaterial to interact with the target site through different receptors, have higher specificity, and accumulate at larger concentrations (Ferro-Flores et al. 2014). Apart from these properties, nanomaterials can potentially increase the specificity and reduce the corresponding side effects (Gupta et al. 2013).

Nanomaterials-based agents can also be efficiently ferried to tumors via passive targeting (relying on the enhanced permeability and retention (EPR) effect noted in many tumors where the chaotic and leaky neovasculature in tumors allows extravasation of nanomaterials from the blood stream and the immature lymphatics within tumors result in augmented retention within them) or active targeting where the surface decoration of nanomaterials allows them to preferentially accumulate within tumors (Prasad 2012; Chen et al. 2016a; Maeda et al. 2000). In principle, nanomaterials-based formulations may improve the conventional imaging and therapeutic efficiency of radionuclides or could be readily tuned to improve specific drawbacks of conventional radionuclide therapy.

#### Classes of nanomaterials

Based on their nature, nanomaterials for radiopharmaceuticals can be classified into organic and inorganic nanomaterials. Organic nanomaterials include liposomes, exosomes, protein-based, polymeric micelles, dendrimers, and polymers, while inorganic nanomaterials include graphene, carbon, iron oxide, silica, gold, and quantum dots. Extensive reviews of nanomaterials investigated on the basis of this classification could be found elsewhere (Kang and Song 2018; Ranjbar Bahadori et al. 2021). Nanomaterials can also be classified based on their dimension into 0D, 1D, 2D, and 3D nanomaterials. 0D nanomaterials include simple nanoclusters(Shamsipur et al. 2016, 2018; Tabrizi et al. 2015), 1D nanomaterials include nanorods and nanotubes (Tsentalovich et al. 2017; Qian et al. 2017; Zhang et al. 2016a; Higginbotham et al. 2010; Nagarajan et al. 2017; Liu et al. 2007), 2D nanomaterials include nanosheets and nanodiscs (Chen et al. 2016b; Farzin et al. 2016; Hiramatsu and Hori 2010), and 3D nanomaterials include nanospheres, nanoshells, and nanocages. (Ding et al. 2015; Lee et al. 2013b; Kim et al. 2017). 0D nanomaterials help transport high doses of diagnostic and therapeutic agents to cancer sites. 1D and 2D nanomaterials have been used for imaging and therapy exploiting either the EPR effect or active targeting, and either as standalone agents or as coupling agents to external activation strategies. 3D nanomaterials have been deployed for imaging and therapy applications that harness their potential for controlled drug delivery, stimuli-responsiveness, selective uncaging, and other such tunable properties that are embedded within the 3D structure design. Overall, when nanomaterials are repurposed as nano-radiopharmaceutical agents, they can go beyond just serving as delivery agents with high radionuclide loading and tumor-specificity to expand the repertoire of functionalities to serving as delivery platforms that exploit distinct biological processes and transport mechanisms. Based on nanomaterials’ dimensions, more extensive descriptions could be found elsewhere (Farzin et al. 2018).

#### Yield, purity, and stability of nano-radiopharmaceuticals

Radiochemical yield is the amount of activity in the product expressed as a percentage of starting activity (Coenen et al. 2018). Radiochemical purity refers to the percentage of the total radioactivity present in the desired chemical form in a radioactive pharmaceutical (Luebke et al. 2000). A high radiochemical purity indicates the absence of any other radioactive element, such as the presence of ^99m^TcO_4_^1−^ and ^99m^TcO_2_ in a^99m^Tc radiolabeled peptide as radiochemical impurities. On the other hand, presence of another radionuclide in the sample, such as ^99^Mo within the ^99m^Tc sample, is an example of radionuclide impurity. Radiochemical stability refers to the strength of the nanomaterial–radionuclide interaction after the radiolabeling of the nanomaterial. It is usually measured *ex-vivo*, providing *in-vivo* conditions. This criterion also accounts for the leakage of radionuclides from nanomaterial.

#### Route of nano-radiopharmaceutical administration

Nanomaterial-based radiopharmaceutical agents can have various routes of administration, e.g., intravenous, intra-tumoral, convection-enhanced delivery, via inhalation, and/or intraperitoneal. The mode of administration depends on the type of tumor. For example, inhalation methods are ideally suited for lung cancer (Muralidharan et al. 2015), intraperitoneal injections for ovarian cancer (Pasqua et al. 2013), convection-enhanced delivery for brain tumors (Fatouros et al. 2006), and intra-tumoral for superficial tumors like sarcomas. Intra-tumoral injection has the advantage of bypassing elimination by the reticuloendothelial mononuclear phagocytic system in the circulation, liver, and spleen. Such uptake can not only reduce tumor accumulation but also potentially increase toxicity. Thus, intra-tumoral administration helps in direct deposition of nanomaterials to the tumor site, lowering toxicity and increasing efficacy (Sinha et al. 2015).

Intravenously administered nanomaterials usually target the tumor site through passive targeting via the EPR effect discussed further in Section “Passive targeting and the EPR effect”. However, given the lack of predictability of the extent of EPR predominance within many human tumors, strategies like thermal stimuli are used to improve efficacy. Herein, the thermal stimulus is first applied to tumor site to enhance extravasation of the intravascular nanoparticle into the tumor resulting in greater accumulation and greater efficacy of the radiolabeled nanomaterial (Islam et al. 2022; Wu 2021; Fang et al. 2020).

#### Nano-radiopharmaceuticals purification and quality control

The presence of various organic solvents, precursors, reagents, etc. within a radiopharmaceutical agent makes it crucial to purify the agent. Apart from considering the limiting factors like radiochemical purity and radiochemical yield, the selection of an appropriate method for radiopharmaceutical purification requires attention to parameters like molecular weight, the half-life of the radionuclide, stability of radionuclide in the mobile phase, the charge of the molecule, lipophilicity, etc. (Nawaz et al. 2017; Mueller et al. 2016). Different purification methods employed are cartridges, High-Performance Liquid Chromatography (HPLC), solid-phase extraction, size-exclusion chromatography, ion-exchange chromatography, liquid–liquid extraction, microfluidics, and microliter droplet technology (Boudjemeline et al. 2017; Wang et al. 2017). A more extensive review of parameters essential for radiopharmaceuticals purification and factors limiting their purification are reviewed elsewhere (Molavipordanjani et al. 2019).

Purification is usually achieved using simple and fast disposable cartridges which can be easily combined with Thin Layer Chromatography (TLC)-based radio-agent purification (Serdons et al. 2008). Another method employs HPLC which provides a better resolution apart from analyzing and separating chemically similar precursor compounds. However, this technique is complicated, time-consuming, and cannot be employed for radionuclides with a short half-life (Matesic et al. 2017). Solid phase extraction is generally utilized midway through radiopharmaceutical preparation and includes normal and reverse solid phase extraction. Size exclusion chromatography is employed during the radiolabeling step, helping remove the excess chelator. Ion exchange chromatography includes cation and anion exchange chromatography which helps to separate simple ions and complex charged molecules from radiopharmaceutical agents. Liquid–liquid extraction is a fast and effective method working on the principle of partition coefficient for separating impurities from radiopharmaceutical agents where the agent is loaded between two immiscible phases (organic solvent and aqueous phase).

The quality control of radiopharmaceutical agents is complicated due to the involvement of radioactive material and is generally performed on the day of administration (Shukla et al. 2013). Furthermore, due to the short lives of some radionuclides, various radiopharmaceutical agents may not have time to undergo all steps of the quality control process (European Directorate for the Quality of Medicines HealthCare 2023). However, the quality control aspects of radiopharmaceutical agents which require maximum diagnostic or therapeutic capacity to lower unnecessary radiation exposure (Saha 2018) also involve biological, physiochemical, pharmaceutical, and toxicity tests extensively reviewed elsewhere (Ekinci et al. 2022). These radiopharmaceutical agents' quality and safety tests involve TLC, HPLC, Ultra Performance Liquid Chromatography (UPLC), mass spectrometry, gas chromatography, etc. with each technique having associated advantages and disadvantages. TLC is fast, precise, cost-efficient, and straightforward that helps detect all the radiolabelled compounds. HPLC, on the other hand, provides better resolution as it uses electrochemical methods, evaporative light scattering, fluorescence spectroscopy, UV–Visible spectroscopy, etc. helping in ensuring chemical and radiochemical purity simultaneously (Ory et al. 2015). UPLC, compared to HPLC, has a shorter analysis time and better sensitivity (Franck et al. 2009; Ha et al. 2017). Mass spectrometry and gas chromatography are specifically useful when purification requires the removal of heavy metals or organic solvents as it helps in the quantification of removed impurities after purification. Microbiological purity is also an essential criterion required for intravenously administered radiopharmaceutical agents. It is usually achieved by direct inoculation and incubation of radiopharmaceutical agents in specific media or involves rapid photometric method or gel-clot for endotoxin quantification (Gee et al. 2008).

## Physiology of tumor targeting

### Passive targeting and the EPR effect

Intravenously administered nanomaterials accumulate preferentially in tumors over normal tissues by exploiting specific features of the tumor, primarily angiogenesis and tumor microenvironment that distinguish it from the vasculature and microenvironment of normal tissues (Liu et al. 2021). In the tumor, angiogenesis is the formation of a new vascular system that arises from the original vascular system as a result of the cancer cells outgrowing and outstripping the preexisting vascular supply as their needs for oxygen and nutrients increase with unrestrained growth. Blood vessels in this newly formed vascular system have abnormal morphological and physiological characteristics. Moreover, the newly (and somewhat hurriedly) formed blood vessels are immature, chaotic, and have discontinuous endothelia with large fenestrations and pores between endothelial lining cells and lack intact basement membranes. These discontinuities in endothelial cells, also called vascular gap openings, are characteristic of tumor sites and the pores are 10–100 times larger than the regular vascular system, 40–200 nm in many instances, making them ideal for extravasation of nanomaterials. On the flip side, the tumor microenvironment is equally immature and inadept at clearing extravasated nanomaterials out of the tumor via well-organized lymphatic channels. This further amplifies nanomaterial accumulation in the tumor. Collectively, this physiological phenomenon of increase uptake and reduced clearance, the so-called EPR effect, allows passive accumulation of nanomaterials in tumors without similar levels of accumulation in adjacent normal tissues (Park et al. 2016; Yang and Gao 2017).

The term EPR was coined in 1986 by Hiroshi Maeda and colleagues (Matsumura and Maeda 1986). The highly permeable vascular system of a tumor to various macromolecular compounds can be exploited to design and develop various anti-tumor agents (Duncan 1999; Torchilin 2011). Specific pathophysiological features of solid tumors include: a) several neo-vascularizations and structural and functional abnormalities in blood vessels (Hori et al. 1993; Benjamin et al. 1999; Suzuki et al. 1981), b) elaboration of a host of inflammatory and neoangiogenic factors (cytokines, chemokines, etc.) that also contribute to chemotaxis of immune cells and macromolecules (Maeda et al. 2000; Wu et al. 1998, 2001, 2002), c) the lack of an efficient lymphatic drainage system (Maeda et al. 2000; Matsumura and Maeda 1986; Leu et al. 2000).

In essence, taking advantage of the EPR effect is considered passive targeting or para-cellular targeting. Here, the nanomaterial accumulates within the tumor due to its poor vasculature, irregular epithelium with vascular gap openings, and poor lymphatic drainage and localizes within the intercellular extravascular space (Kumari et al. 2016; Masood 2016; Mahato 2017). In passive targeting, the targeting agent travels to the tumor site via the blood stream, enters the tumor site through leaky blood vessels and various factors released by the tumor, and accumulates at the tumor site due to poor drainage. In this type of targeting, the radiopharmaceutical's success depends on the circulation time. Various polymer-radiopharmaceutical complexes have been investigated for passive targeting (Chen et al. 2016c; Ulbrich et al. 2016).

### Trans-cellular targeting

Trans-cellular targeting is also called active targeting. This involves biological communication between radiopharmaceutical agents and tumor surfaces. In active targeting, the radionuclide is bound to vectors (antibodies, peptides, etc.) specific to the tumor with or without the use of chelators. Active targeting recognizes specific cells in the tumor microenvironment, and is ideally suited for tumors with low permeability (Hansen et al. 2015). However, a recent metanalysis of active targeting strategies used for nanomaterial transport to tumors suggested that less than 0.7% of injected dose accumulates within the tumor (Wilhelm et al. 2016). On the other hand, one potential advantage of active targeting is the cellular subtype localization and/or subcellular localization of the nanoparticle (Huang et al. 2010). When nanomaterials are decorated with moieties that help them bind to specific receptors on the cancer epithelial cell vs. cancer fibroblast vs. other stromal cells, the nanomaterial may not accumulate in much higher amounts within the tumor but it may accumulate preferentially in the targeted cell in much higher amounts than when the nanomaterial is untargeted. Alternatively, the nanomaterial may be internalized within the cell (via receptor-mediated internalization) when actively targeted vs. untargeted and this might provide a key advantage, say with Auger emitters that are more effective when closer to nuclear DNA. This targeting seems to help overcome multidrug resistance and can be used to cross the blood–brain barrier (Salahpour Anarjan 2019; He et al. 2020; Lin et al. 2016; Nag and Delehanty 2019).

## Methods of radiolabeling nanomaterials

### Direct radiolabeling

**Fig. 8:**
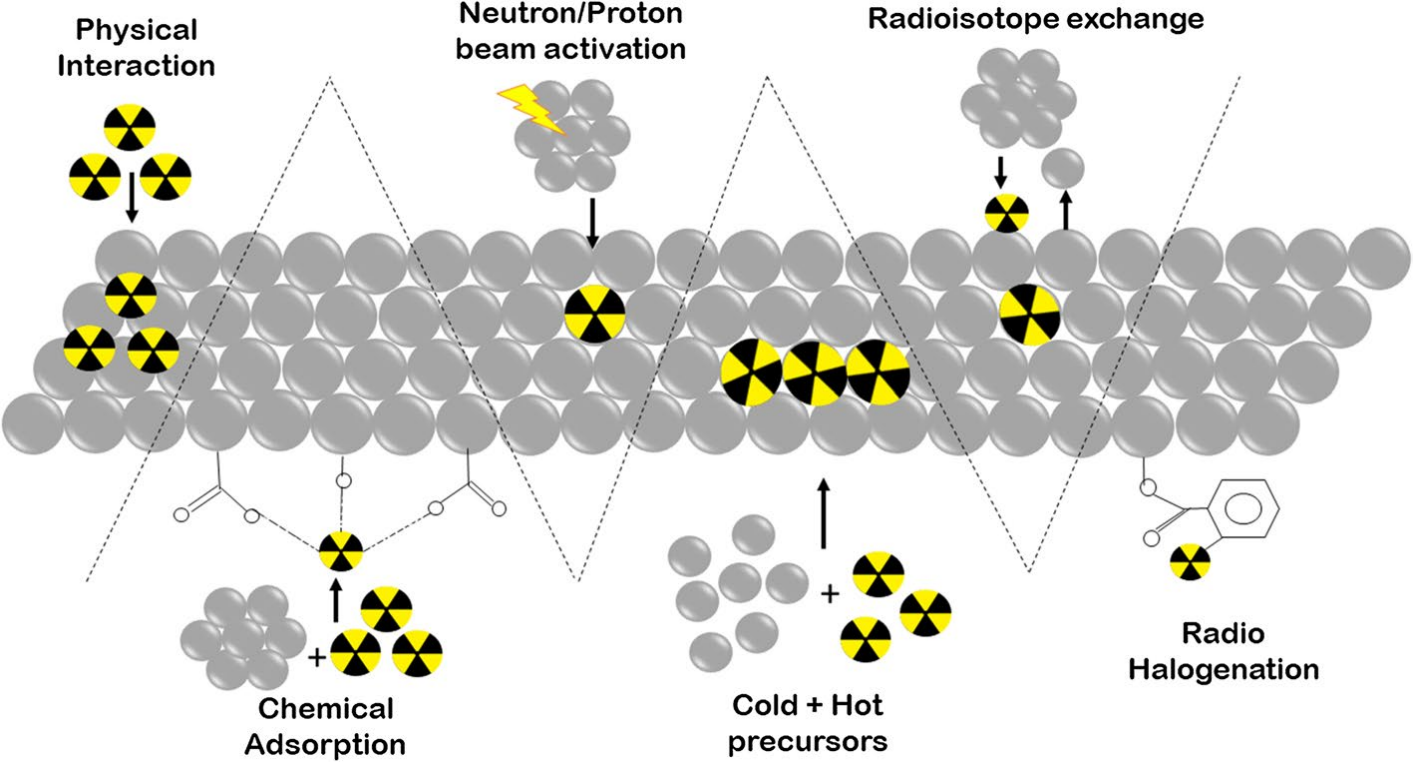
Schematic representation of various methods of direct radiolabeling nanomaterial, i.e., physical interaction, chemical adsorption, neutron/proton beam activation, coprecipitation, radioisotope exchange, and radiohalogenation

Direct radiolabeling (non-chelator-based radiolabeling) does not require a chelator for radiolabeling nanomaterials. In this, the radionuclide is directly incorporated on the surface or in the core of the nanomaterial. Knowledge about nanomaterials and radionuclides is essential in this type of radiolabeling. Many non-metallic radionuclides (e.g., ^18^F, ^11^C, and ^131^I) use this technique. The significant advantages of this technique are that it is straightforward, time-efficient, has a smaller number of reaction steps, retains higher integrity and stability of the nanomaterial, and obviates the need for bulky chelators that link the radionuclide to the nanomaterial thereby improving in-vivo activity (Goel et al. 2017). This radiolabeling method is less complex as the radionuclides follow common nanomaterial integration steps. Some of the commonly employed radiochemical reactions to incorporate radionuclides within nanomaterials includes halogenation, chemical adsorption, coprecipitation, proton or neutron beam activation, radioisotope exchange, and/or physical interactions as depicted in Fig. 8.

#### Physical interaction

This technique takes advantage of the physical properties of the nanomaterial. The radionuclide is either attached to the cavity, defect, or groove of the nanomaterial as depicted in Fig. 8. Sometimes, weak electrostatic forces help in radionuclide and nanomaterial interaction (Lemaître et al. 2022). This method is also called physical adsorption or physisorption. However, this approach has limited applications due to the poor stability of interaction, incomplete knowledge of radiolabeling mechanism, and/or lack of appropriate nanomaterials specific to this technique. One of the most common examples of this technique is the incorporation of ^64^Cu in the cavity of single-wall carbon nanotubes (Cisneros et al. 2014).

#### Chemical adsorption

This technique exploits the chemical properties of nanomaterials for direct adsorption of a radionuclide on the nanomaterial’s surface as depicted in Fig. 8 (Roldan Cuenya et al. 2010; Králik 2014). The adsorption occurs by forming coordination bonds between chemical groups like –PO_3_H_2_, –SH, -NH_2_ or –OH of nanomaterial with the radionuclide. Use of this technique hinges on the affinity and stability of the chemical interaction. As this technique requires high temperature, it is vital to ensure that the properties of nanomaterial are not affected. In 2013, this technique was first utilized for chemical adsoption of radionuclides ^71^As, ^72^As, ^74^As, and ^76^As on the surface of iron oxide nanoparticles (Chen et al. 2013). Other examples include chemisorption of ^64^Cu, ^89^Zr, and ^111^In on the surface of feraheme/ferumoxytol nanoparticles (Boros et al. 2015), ^68^ Ga, ^111^In, ^177^Lu, ^90^Y, and ^89^Zr on silanol groups of silica nanoparticles (Shaffer et al. 2015), and ^64^Cu on thiol group of silica nanoparticles (Shaffer et al. 2016).

#### Radio-halogenation

Radio-halogenation is widely used for radiolabeling nanomaterial with iodine radionuclide (radio-iodination). This primarily employs the electrophilic substitution reaction; however, nucleophilic substitution reaction is also employed. The tyrosine, histidine, or other moieties on the surface of nanomaterial are routinely radiolabeled as depicted in Fig. 8. Iodogen, iodobeads (Kumar and Woolum 2021), chloramine-T (Coenen et al. 2006), or the Bolton-Hunter reagent act as oxidizing agents that react with the iodine anion for electrophilic substitution in the ortho-position of phenol (of tyrosine). Iodobeads, iodogen, and chloramine-T generally radioiodinate tyrosine or histidine moieties, while the Bolton-Hunter reagent can radiolabel nearly all free amino groups of nanomaterials. Radionuclides frequently used include ^124^I, ^125^I, and ^131^I. Radioiodination is a quick method with a high yield; however, poor stability of this method has been reported (Kostiv et al. 2017; Black et al. 2014). Apart from radioiodination, radio-bromination (Liu et al. 2009a; Almutairi et al. 2009) and radio-fluorination (Guerrero et al. 2012; Mauro et al. 2015; El-Marakby et al. 2017; Akca et al. 2014) are also used for radio-halogenation. Moreover, for radio-halogenation, click chemistry and other reactions can also be utilized (Simone et al. 2016; Ghiassian et al. 2019; Wu et al. 2019; Jeon et al. 2015; Keliher et al. 2017; Reibel et al. 2015; Wagener et al. 2018; Meyer et al. 2016). Click reactions majorly employed for nanomaterials radiolabeling are cycloaddition of azide to terminal alkyne with copper as catalyst, and Diels–Alder-based cycloaddition between tetrazine and trans-cyclooctene without copper as a catalyst (Meyer et al. 2016).

#### Radioisotope exchange

In this technique, an element present on the nanomaterial is replaced with a radionuclide as depicted in Fig. 8. This is a simple, efficient method; however, only limited nanoparticle to radioisotope exchange combinations are effective. Homogenous radioisotope exchange involves using different isotopes of the same element, while heterogeneous radioisotope exchange involves replacement between different elements. The homogeneous radioisotope exchange has been used for radiolabeling up-converting nanoparticles (Liu et al. 2011; Zhou et al. 2011). The heterogeneous radioisotope exchange usually produces high yield and purity under mild conditions. It has been employed in radiolabeling iron oxide nanoparticles with ^68^ Ga, quantum dots with ^64^Cu and ^68^ Ga, and up-converting nanoparticles with ^153^Sm (Sun et al. 2013, 2014a; Tang et al. 2019; Israel et al. 2015).

#### Neutron or proton beam activation

This technique is applicable and specific to inorganic nanomaterials. Here, high energy proton or neutron beams bombard the nanomaterial causing particular atoms of the nanomaterial to undergo a nuclear reaction as depicted in Fig. 8. These nanomaterial atoms then produce radionuclides (Sun et al. 2015). The significant advantage of this technique is high control over the location of the radionuclide within the nanomaterial. However, this technique requires specific nuclear reactors or accelerators which may not be readily available. Moreover, the use of a high-energy proton or neutron beam may affect biologically synthesized nanomaterials, limiting the use of this technique to inorganic nanomaterials. Some examples of production of radionuclides using this technique include (a) the ^18^O(p,n)^18^F nuclear reaction where 16 MeV protons bombard ^18^O-enriched Al_2_O_3_ nanoparticles and convert ^18^O to ^18^F (Pérez-Campaña et al. 2012), (b) the ^16^O (p,α)^13^N proton activation reaction for the conversion of ^18^O to ^13^N in Al_2_O_3_ nanoparticles (Pérez-Campaña et al. 2013), (c) the ^165^Ho(n,γ)^166^Ho nuclear reaction where a neutron beam is targeted on holmium-based garnet magnetic nanoparticles (Nayak and Lahiri 1999; Nijsen et al. 2001), (d) the ^152^Sm(n,γ)^153^Sm nuclear reaction, and (e) the ^158^Gd(n,γ)^159^Gd nuclear reactions for boron nitride nanotubes (Silva et al. 2020; Munaweera et al. 2015).

#### Radioactive coprecipitation

This technique is specific for inorganic nanomaterials. Here, a mixture of nanomaterial reagents (cold precursors) and a mixture of reagents containing radionuclide (hot precursors) reacts to produce radiolabeled nanomaterial in a single step as depicted in Fig. 8. This method is also called hot-and-cold mixing. The reaction protocol is fast, straightforward, and time-efficient and is widely used in radiolabeling. The nanomaterials are radio-chemically doped during their synthesis. The trace levels of hot precursors coprecipitate with the nanomaterial reagents, leading to the incorporation of radionuclide in the crystal lattice of the nanomaterial (Lamb and Holland 2018). The doping allows the synthesis of nanomaterials of desired integrity while providing stability in radiochemical labeling. This technique is highly used in homo-radionuclide doping, where the same element is present in the nanomaterial and the radionuclide. For example, gold nanoparticles can readily be doped with ^195^Au, ^198^Au, or ^199^Au (Kreyling et al. 2018; Zhao et al. 2016; Chanda et al. 2010; Wang et al. 2013).

For efficient yield, important parameters to consider are the ionic radii of hot and cold precursors, same ionic charge on hot and cold precursors, high solubility between cold and hot precursors, physico-chemical properties of the radionuclide, and controlling the ionic strength of the nanomaterial reaction medium for nucleation and synthesis of nanomaterial. As radionuclides are usually present in an aqueous medium, this technique can be applicable to nanomaterial synthesis in water. Apart from gold nanoparticles, other examples of radiolabeling via this technique include iron oxide nanoparticles doped with ^225^Ac, ^64^Cu, ^59^Fe, ^68^ Ga, ^111^In (Weissleder et al. 1989; Pouliquen et al. 1989; Chouly et al. 1996; Pellico et al. 2016; Zolata et al. 2016; Wong et al. 2012; Cędrowska et al. 2020; Zeng et al. 2014), quantum dots doped with ^109^Cd, ^64^Cu, ^125m^Te (Sun et al. 2012; Kennel et al. 2008; Guo et al. 2015), silver nanoparticles doped with ^131^I (Sakr et al. 2018), cerium oxide nanoparticles doped with ^141^Ce, ^65^Zn (Yang et al. 2013a), and up-converting nanoparticles doped with ^153^Sm, ^90^Y (Yang et al. 2013b).

### Chelator-based radiolabeling

Certain radionuclides do not form stable bonds with vectors like antibodies and peptides. They often require a chelator to provide stability. A chelator is a molecule containing a ligand (typically organic) bonded to a central metal atom at two or more points, thereby producing highly stable complexes with radionuclides. Most metallic radionuclides cannot form strong bonds with organic compounds and require chelators (Price and Orvig 2014). Metallic radionuclides, also called radiometals, include the commonly used imaging agents ^64^Cu, ^89^Zr, and ^99m^Te. For radiometals to form stable conjugates with nanomaterials for proper *in-vivo* biodistribution, it is crucial to understand the coordination chemistry of the radiometal. Some important coordination chemistry factors are atomic number, atomic radius, atomic charge, coordination number, and geometry preferences. Other factors include the hardness of the radiometal as assessed by the Pearson acid–base concept, hard or soft donor atoms of the chelator, and suitable electronic properties that improve the kinetic energy of the radionuclide-chelator complex (Price and Orvig 2014; Park and Kim 2013).

The thermodynamic stability of the chelator contributes to its ‘chelating effect’. A chelator is considered to have a higher chelating effect if complex formation results in increased entropy. A polydentate chelator forms stable complexes with radiometals as compared to monodentate ligands. They, thus, have a better chelating impact due to higher entropy. The polydentate ligands can be further classified into acyclic and macrocyclic. Acyclic chelators, also called linear chelators, are highly flexible and result in a quick complexation with the radiometals. Macrocyclic chelators are rigid due to their pre-organized structure, resulting in lower complexation kinetics but higher complexation stability (macrocyclic effects). Due to their lower complexation kinetics, macrocyclic chelators have longer reaction rates and require higher temperatures for reactions (Price and Orvig 2014; Sneddon and Cornelissen 2021). The longer reaction rates and higher temperatures may negatively affect certain nanomaterials (protein-based, exosomes). Bifunctional chelators are used to overcome this problem.

An ideal chelator has rapid complex formation under mild conditions (pH, temperature), exhibits high kinetic stability, harbors a high chelating effect, and displays high thermodynamic stability. The half-life of radiometals and pharmacokinetics of nanomaterials also play a role in the time duration of radiometal–chelator–nanomaterials complex stability (Sneddon and Cornelissen 2021; Hu and Wilson 2022).

#### Bifunctional chelators

**Fig. 9:**
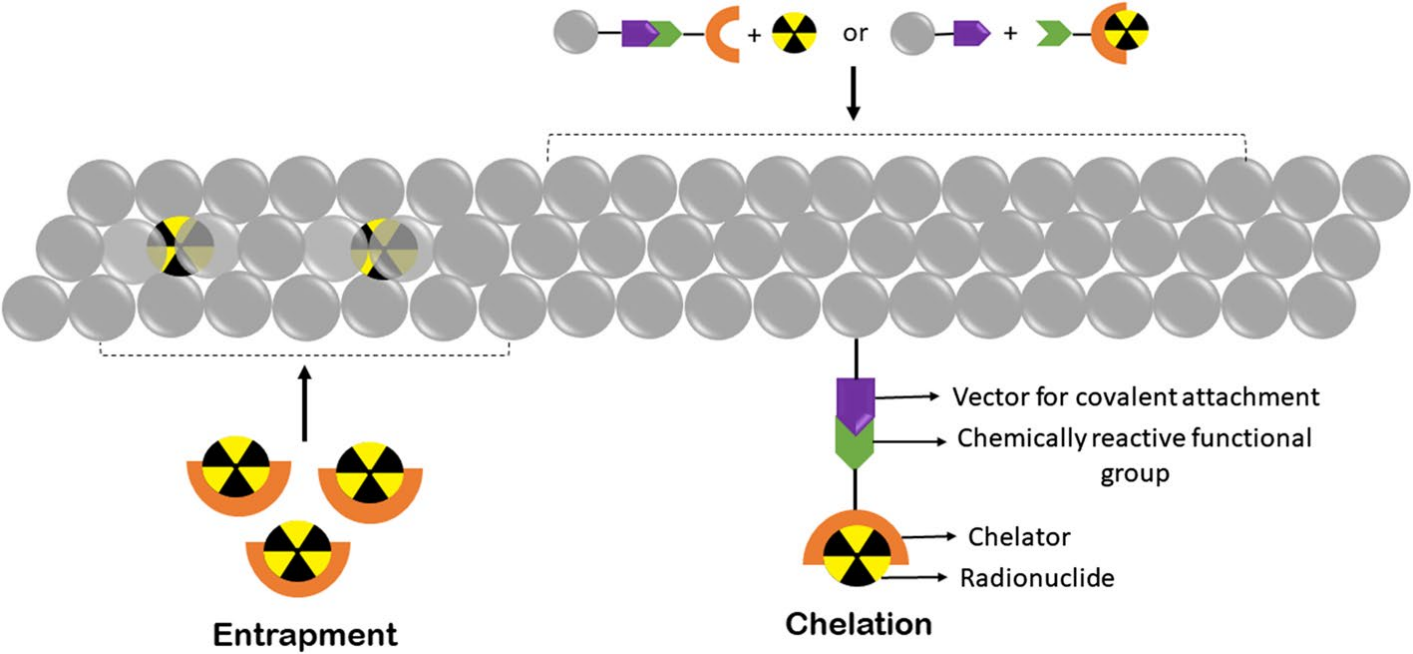
Schematic representation of some methods of indirect radiolabeling of nanomaterials, i.e., entrapment and use of bifunctional chelators

Bifunctional chelators play an important role in the radiochemistry of radiometals. They are widely used in the radiolabeling of nanomaterials. Bifunctional chelators consist of a chelating group that binds to a radionuclide and also has a functional moiety that attaches to the functional group of a nanomaterial, protein, or other vectors as depicted in Fig. 9 (Price and Orvig 2014). Thus, a bifunctional chelator undergoes two conjugation reactions by binding a radionuclide and also covalently linking a nanomaterial (Hermanson 2013). The functional group on nanomaterials may be present intrinsically or synthetically created on the nanomaterial to facilitate conjugation. The functional groups on nanomaterial, namely amine, thiol, carboxylic acid group, etc. form conjugates with bifunctional chelators.

In amine conjugation, an amide bond is formed when the amine group of the nanomaterial (dendrimers or lipid nanomaterials) reacts with activated ester groups or cyclic anhydrides of the chelator. In a different amine conjugation, isothioureas can also be formed when the amine group of the nanomaterial reacts with the aryl isothiocyanate of the chelator. In carboxylic acid conjugation, the amine group of the chelator binds to the carboxylic acid group of the nanomaterial (polymer-based, protein nanomaterials) using carbodiimide coupling reagents (e.g., EDC) or binds to the thiol group of nanomaterial using maleimides (Hermanson 2013). Click chemistry can also be used for fast, high-yield reactions. The major reactions in click chemistry are copper-catalyzed, or catalyst-free azide-alkyne and Diels–Alder cycloaddition reaction between a tetrazine and a trans-cyclooctene (Meyer et al. 2016; Zeglis and Lewis 2011; Ramogida and Orvig 2013).

#### Entrapment of radionuclide within nanomaterials

This method is entirely dependent on the reagents involved in nanomaterial synthesis. Here, a radiometal first binds to a specific chelator. This radiometal–chelator complex is then used with other reagents used in the nanomaterial synthesis. Thereby, the radiometal gets trapped within the nanomaterial producing a radiolabeled nanomaterial as depicted in Fig. 9. This method is suitable for polymeric micelle-based nanomaterials that have lipophilic pockets for entrapping lipophilic radiometals (Ferreira et al. 2019). The key factors for this method of radiometal–nanomaterial production require compatible nanomaterials, nanomaterials with short synthesis time, or radionuclides with long half-lives. It is critical to maintain the stability of the radiometal–nanomaterial complex during synthesis and subsequent purification of nanomaterials in this technique (Ranjbar Bahadori et al. 2021).

#### Ionophore-based radiolabeling

This technique is specific for vesicle-based nanomaterials that have lipid bilayer membranes (e.g., liposomes and exosomes). Here, the ionophore reversibly binds to a radionuclide and forms a lipophilic complex capable of crossing the lipid membrane (Steinbrueck et al. 2020). The radionuclide–ionophore complex crosses the lipid bilayer, enters the core/cavity of the nanomaterial, and the radionuclide trans-chelates from ionophore to the nanomaterial which houses chelating molecules to entrap the radiometal with high affinity within the core. Non-macrocyclic, low denticity chelator ionophores are used as they can form a low-affinity metastable complex with the radiometal. The chelating molecules of the nanomaterial might be intrinsic (proteins or nucleic acids in exosomes, drugs in liposomes) or added as reagents during nanomaterial synthesis (Aranda-Lara et al. 2020). Important factors to consider in this method are the quantity of radiometal–ionophore complex loaded in the nanomaterial (loading efficiency), instability of the radiometal–ionophore complex to enhance the release of radiometal in the nanomaterials, high affinity of the radiometal with the chelating molecules of the nanomaterial, and the efficiency of these interactions in in mild, physiological conditions (Man et al. 2019).

#### Remote loading of radionuclides

This technique is similar to ionophore-based radiolabeling albeit without an ionophore bound to a chelator. The radionuclide of choice is lipophilic enough to cross the lipid membrane of the nanomaterial, lipophobic enough to prevent radiometal solubility, and stable in the vesicle core. Moreover, functional groups on the radionuclide develop a charge in the aqueous environment of the nanomaterial core, aiding in entrapment with nanomaterial chelators (Kang and Song 2018; Ge et al. 2020; Pérez-Medina et al. 2020; Rhim et al. 2015).

## Applications of radiolabeled nanomaterials

### Radionuclide imaging and therapy

This application of radiolabeled nanomaterials uses α, β-, or Auger electron emitters to mediate tumor destruction (Qaim 2001; Yeong et al. 2014). The particulate radionuclides may be bound to a vector, like a peptide or antibody via direct or indirect radiolabeling and conjugated with the nanomaterial. Some examples of radiolabeled nanomaterial for breast cancer imaging and therapy are ^99m^Tc-labeled nanoemulsion of a natural compound, lapachol, for imaging its biodistribution (Mendes Miranda et al. 2021), ^99m^Tc(CO)_3_-labeled magnetic nanoparticles with D-penicillamine as a chelating agent for imaging and therapy (Özyüncü et al. 2016), ^99m^Tc-labeled nanostructured lipid carrier loaded with α-tocopherol succinate and doxorubicin for imaging of this combination chemotherapeutic agent (Fernandes et al. 2018), ^177^Lu-labeled anti-HER2 nanobodies for imaging and therapy (D’Huyvetter et al. 2012), ^68^ Ga-labeled bimetallic silver-gold nanoparticles synthesized by chemical reduction using tryptophan for imaging (Katifelis et al. 2020), ^111^In-labeled multifunctional superparamagnetic iron oxide nanoparticles conjugated to doxorubicin and trastuzumab for dual-modality imaging and alternating magnetic field hyperthermia (Zolata et al. 2016), and ^111^In-labeled PEGylated thermally oxidized porous silicon nanoparticles for imaging (Lumen et al. 2019). In a similar vein, ^211^At-labeled PEGylated gold nanoparticles have been used as potent alpha particle therapeutic agents in pancreatic cancer and gliomas (Kato et al. 2021) and ^99m^Tc-radiolabeled paclitaxel loaded biodegradable PEGylated polymeric ε-caprolactone nanoparticles for imaging neuroendocrine pancreatic tumors (Dubey et al. 2012). In another approach, ^177^Lu-labeled fourth-generation PAMAM dendrimers with gold within their cavities were decorated with bombesin and folate to target gastrin-releasing peptide receptors and folate receptors, respectively, on lung cancers to facilitate seamless imaging and therapy of these tumors (Wang et al. 2022) and ^131^I-labeled silver nanoparticles synthesized via a green chemistry method using the natural product shikonin as the reducing and capping agent have been explored for imaging of lung cancers (Fayez et al. 2020). As is evident from these early examples of use of radiolabeled nanomaterials, initial development was focused mostly on radiolabeling of nanoconstructs merely to facilitate visualization of the construct in vivo (for biodistribution and pharmacokinetic analyses) and subsequent formulations have increasingly explored the use of theranostic radiolabeled nanomaterials for diagnostic and therapeutic purposes.

### Radionuclide-based diagnostics

Radionuclides with γ and β + emissions are readily visualized by SPECT and PET imaging to facilitate diagnosis and serial longitudinal non-invasive monitoring of the natural history of cancer (de novo or upon treatment), as discussed in Section “Radionuclides for Spect and Pet-based diagnostics”. However, the specificity and effectiveness of diagnostic radionuclides can be improved using nanomaterials, as discussed in Section “Methods of Radiolabeling Nanomaterials”.

Radiolabeling of nanomaterials is used extensively to assess the biodistribution of newly formulated agents in-vitro and biodistribution and pharmacokinetics in-vivo (Tang et al. 2016).

### Multimodal imaging

Multimodal imaging makes use of an imaging technique in combination with nuclear imaging, potentially allowing increased resolution, greater sensitivity, and provision of complementary anatomical and physiological information. Outlined below are some examples of this multimodal imaging approach.

#### Radiolabeled nanomaterial-based PET/MRI multimodal imaging

MRI provides anatomical, physiological, and molecular information and is routinely used by radiologists in the clinic to diagnose cancers and monitor the efficacy of therapeutic interventions (Vallières et al. 2015; Kasivisvanathan et al. 2018; Valerio et al. 2015; Li et al. 2015a). However, it may have poor sensitivity (Goel et al. 2017; Liu et al. 2015). Therefore, radiolabeled nanomaterial-based PET combined with MRI can provide higher sensitivity and accuracy (Goel et al. 2017; Shao et al. 1997). There are two nanomaterial-based MRI contrast agents that could be radiolabeled—those that provide contrast on T1-weighted images and those that provide contrast on T2-weighted images. T1 contrast agents provide darker images and include paramagnetic inorganic nanoparticles radiolabeled with Gd or Mn. T2 contrast agents provide brighter (whiter) images and include superparamagnetic iron oxide-based nanomaterials radiolabeled with ^99m^Tc.

Gd-radiolabeled inorganic nanomaterials have been extensively studied (Liu et al. 2014; Cheng et al. 2017; Abou et al. 2013; Cao et al. 2017). For example, ^64^Cu-DOTA-GDVO4 with Asp-Gly-Glu-Ala (DGEA) peptide recognizes integrin α_2_β_1_ on human prostate cancer (Hu et al. 2014a). Similarly, ^99m^Tc-radiolabeled manganese oxide-based silica nanoparticles have also been studied (Gao et al. 2016). Surface polymer-coated superparamagnetic iron oxide nanoparticles are extensively employed (Sun et al. 2016; Madru et al. 2012; Liu et al. 2009b; Misri et al. 2012; Kim et al. 2013). For example, ^99m^Tc-labeled Fe_3_O_4_ nanoparticles have been synthesized to target glutathione on cancer cells (Gao et al. 2017).

Sometimes, SPECT/MRI is employed to provide imaging with better contrast, sensitivity, and resolution. For example, ^125^I-(cRGD)2-IONPs contain iron oxide nanoparticles and peptides that target the α_v_β_3_ and α_v_β_5_ receptor (Sun et al. 2019).

#### Radiolabeled nanomaterial-based SPECT/CT multimodal imaging

Computed tomography (CT), widely used in clinics, uses X-rays and provides contrasting images of tissue of interest. It is affordable, with excellent spatial resolution, and accurate anatomical data. Materials such as Ag, I, Au, Ta, and Bi provide stronger absorption of X-rays and serve as efficient contrast agents (Kim et al. 2007; Dong et al. 2014; Liu et al. 2013a). SPECT is commonly coupled with CT in clinical practice. Again, piggybacking radiolabeled nanomaterial use for imaging onto a modality that is widely available in clinical settings, affordable, and many radiologists are familiar with already allows more widespread dissemination and utilization. For example, dendrimer-entrapped gold nanoparticles radiolabeled with ^99m^Tc and functionalized with folic acid have been used in dual-modality SPECT/CT imaging (Li et al. 2016). Herein, the nanoprobes have better accumulation at the tumor site, and folic acid functionalization aids active targeting. Similarly, hyaluronic acid-coated silver nanoparticles radiolabeled with ^99m^Tc have also been developed (Zhang et al. 2016b).

#### Optical imaging

Optical imaging is a highly sensitive technique that helps study molecular-level physiological changes and is useful for small tumor detection (Qiao et al. 2015; Liu et al. 2013b; Li et al. 2017, 2018, 2019; Hu et al. 2017). It can be broadly classified into luminescence and fluorescence imaging. In fluorescence imaging, excitation light excites the reporter group, which then emits energy when it returns to the ground state. Some common reporter groups employed are dyes (Kumar et al. 2008; Pérez-Medina et al. 2014), proteins (Calvo-Alvarez et al. 2018; Jiang et al. 2017), quantum dots (Selvan et al. 2005; Gao et al. 2004; Michalet et al. 2005; Yong 2012; Sun et al. 2014b), and up-conversion nanoparticles (Yang et al. 2012a; Wang and Liu 2009). Luminescence imaging uses light produced during a chemical reaction to visualize tumors, gene expression, and other diseases (Black et al. 2014; Sun et al. 2014a; Guo et al. 2015; Qiu et al. 2018).

Optical imaging is widely used in biological studies (Black et al. 2016). Its sensitivity, specificity, and resolution are further improved via fluorescence-mediated tomography, bioluminescence tomography, and Cherenkov fluorescence imaging. The biggest impediments to routine clinical use are the non-uniform light scattering through tissues, limited tissue absorption, and poor depth of penetration (Yang et al. 2013b; Zhan et al. 2016; Tang et al. 2012). Radiolabeled nanomaterials capable of Cherenkov luminescence imaging, fluorescence imaging, Raman imaging, and up-conversion luminescence imaging can help overcome these challenges of optical imaging technology.

##### Radiolabeled nanomaterial-based PET/Cherenkov luminescence imaging

When a charged particle travels at a speed greater than the phase velocity of light in a dielectric medium of the same radioisotope, it emits electromagnetic radiation called Cherenkov radiation. Some elements with measurable Cherenkov emissions are ^18^F, ^13^N, ^32^P, ^131^I, etc. (Mitchell et al. 2011), which are often employed in diagnostic imaging (Thorek et al. 2014; Spinelli et al. 2013). Nanomaterials (carbon dots, silver nanoparticles, up-conversion nanoparticles, quantum dots) can be employed to improve the low penetration of Cherenkov radiation. Nanomaterials absorb Cherenkov-emitted light and emit longer wavelength near-infrared light (Sun et al. 2014a; Hu et al. 2014b). Cerenkov resonance energy transfer (CRET) could also improve penetration depth and sensitivity (Sun et al. 2014a). Some examples include ^64^Cu incorporated in CuInS/ZnS quantum dots (Pellico et al. 2016) and ^64^Cu-doped gold nanoparticles (Hu et al. 2014b) used in PET/CRET luminescence imaging.

##### Radiolabeled nanomaterial-based fluorescence imaging

Fluorescence imaging can span the spectrum from ultraviolet to visible to near-infrared wavelengths, with infrared wavelengths having better penetration. It is employed in clinics for imaging during tumor removal, distinguishing normal and pathological tissues, and benchmark molecular characteristics of tumors. (Roberts et al. 2011; Liberale et al. 2018; Li et al. 2015b; Jewell et al. 2014). Near-infrared-based fluorescence imaging can be classified into 650–950 nm and 1000–1700 nm based on absorption in human tissues (Chen et al. 2018a; Hong et al. 2017). Both optical windows provide better penetration due to less interference by absorption by naturally occurring fluorochromes in physiological tissues, excellent resolution, and low normal tissue autofluorescence (Chen et al. 2018a; Duan and Liu 2018; Wang et al. 2019; Miao and Pu 2018). Thus, it has found its applications in numerous diagnostics.

Radiolabeled nanomaterials conjugated with fluorescent dyes (Liu et al. 2012) are of value in radiolabeled nanomaterial-based fluorescence imaging. For example, dextran-coated iron oxide nanoparticles radiolabeled with PET isotopes conjugated with near-infrared fluorochromes and a targeting vector have been used for colon carcinoma imaging (Nahrendorf et al. 2010). Similarly, for glioma tumors, chimeric ferritin nanocages with ^64^Cu in their cavity and near-infrared fluorescent dye Cy5.5 and RGD4C on their surface have also been developed for PET/ near-infrared-based fluorescence imaging (Lin et al. 2011). Recently, Cu–In–Se quantum dots containing ^111^In with a nonradioactive ZnS shell were developed for SPECT/fluorescence imaging (Sun et al. 2014b).

##### Radiolabeled nanomaterial-based PET/Raman multimodal imaging

Raman imaging makes use of the Raman scattering principle where molecules excited to higher energy levels produce inelastic scattering of photons. However, typically, there are very few inelastically scattered photons. In 1974, Fleischmann et al. observed that pyridine molecules deposited on the surface of nano-scale rough silver electrodes helped produce Raman signals with high intensity (Fleischmann et al. 1974). Later, Jeanmaire et al. reported that surface roughening with nano-Au, Ag, Cu, etc. has six times better Raman signal (Jeanmaire and Duyne 1977). This technique is called Surface-Enhanced Raman Scattering (SERS). This technique is advantageous in tumor imaging as it is not affected by autofluorescence (Zavaleta et al. 2011) but has limited tumor penetration on the downside. To improve penetration, Wall et al. researched ^68^ Ga-SERS-NPs for combined tumor imaging via PET/SERS (Wall et al. 2017).

##### Radiolabeled nanomaterial-based PET/Up-conversion luminescence imaging

Conversion of light of lower energy to higher energy via energy transfer or multiple photon absorptions is called up-conversion luminescence. It employs up-conversion nanoparticles like lanthanide-doped rare earth nanoparticles and is widely used in tumor imaging (Liu et al. 2013b; Tian et al. 2019; Cheng et al. 2013). Due to poor penetration, up-conversion luminescence is used in conjugation with PET (Yang et al. 2013b; Peng et al. 2013). For example, up-conversion NaGdF4:Yb,Tm,Ca@NaLuF4 core@shell nanoparticles conjugated with anti-HER2 monoclonal antibody and radiolabeled with ^99m^Tc have been used in metastatic lymph node diagnosis (Qiu et al. 2018).

#### Radiolabeled nanomaterial-based photoacoustic imaging

Photoacoustic imaging is non-invasive imaging modality where energy absorbed by tissues exposed to a pulsed laser irradiation is converted to heat; the thermal expansion and contraction of irradiated tissue generates an ultrasonic wave that is detected by a transducer array (Cheng et al. 2014a; Yang et al. 2012b; Doane and Burda 2012; Kircher et al. 2012). Agents of photoacoustic imaging include inorganic nanomaterials, organic nanomaterials, and near-infrared-based molecules (Miao and Pu 2018; Liu et al. 2019; Li and Pu 2019). Coupling PET imaging with photoacoustic imaging of radiolabeled nanomaterials has been used in many in-vitro and in-vivo imaging applications (Cheng et al. 2014b; Zhang et al. 2014; Yang et al. 2017; Jiang et al. 2018; Chen et al. 2018b; Wang et al. 2016a). For example, PEGylated Au-tripods containing RGD peptide and radiolabeled with ^64^Cu have been used to visualize tumors efficiently in the glioblastoma xenograft models. Similarly, ferritin nanocages containing CuS nanoparticle in its cavity radiolabeled with ^64^Cu have been used for PET/photoacoustic imaging in human glioblastoma (Wang et al. 2016a).

#### Radiolabeled nanomaterial-based tri or multi-modality imaging

The advent of radiolabeled nanomaterial-based bi-, tri-, or multi-modality imaging has improved the prospects for complementary diagnostics. For example, radiolabeled nanomaterial-near infrared fluorescence-MRI (NM/nIR/MRI) (Xu et al. 2018; Li et al. 2014), radiolabeled nanomaterial-CT-photoacoustic imaging (NM-CT-PAI) (Wang et al. 2016b), NM-PAI-MRI (Liu et al. 2015; Guo et al. 2018; Chen et al. 2017b; Fan et al. 2014; Jin et al. 2017), and NM-PAI-CT-MRI (Cheng et al. 2016) have been reported.

## Regulation of radiopharmaceuticals

As with all new pharmaceutical formulations marketed as drugs for human use, many steps are involved in regulatory approval of nanoradiopharmaceuticals. The principles of manufacturing are similar to other drugs in that standard operating procedures need to be established for uniform batches of agents with clearly benchmarked physicochemical properties to be synthesized consistently in good manufacturing practice facilities. Scale-up for clinical use will need to be factored into the facility design and establishment. Good laboratory practice toxicological studies will likely be needed in two mammalian species for filing an investigational new drug application for Food and Drug Administration (FDA) clearance in the United States. On occasion, nanomaterials have cleared the FDA as devices rather than as drugs but in the case of nanoradiopharmaceuticals it is more likely that the filing will be as a drug or possibly, a drug-device combination. In either case, some guidance is available from the Nanotechnology Characterization Laboratory of the National Cancer Institute from their experience with extensive testing of over a hundred nanomaterials in preclinical immunological, biochemical, histopathological, and functional toxicity studies. Specific issues may need to be addressed for unique indications. Being relatively new to regulatory review, we anticipate many nanoradiopharmaceuticals requiring multiple angles of review by experts at regulatory agencies, many of whom may also not be proficient with all aspects of safety considerations but with enough experts in the room, it is likely that collective expertise will be available at the FDA review panel. This review will likely provide some framework for the composition and conduct of such a review.

## Outlook

Over the last two decades, here has been a large surge of interest in integrated research at the intersection of nuclear medicine and nanotechnology. This emerging convergent science has the potential to overcome deficiencies and drawbacks in extant radionuclide therapy. Continued growth and expansion of this interdisciplinary domain requires cross training of scientists in these otherwise non-overlapping disciplines. Compilation of a lexicon of the vocabularies, basic underlying principles, and state-of-the art in each of these disciplines could fuel the quest for new discoveries and bench-to-bedside translational possibilities. With such a framework in mind, we have concisely reviewed conventional radiopharmaceutical systems and how nanomaterials integration could be advantageous.

As is evident from such an undertaking is that there are distinct advantages and disadvantages associated with each method of radiolabeling nanomaterials, each imaging modality, and each potential formulation of nano-radiopharmaceuticals. A more wholistic and yet individualized approach that maximizes the advantages of each technique or approach could catalyze the emergence and adoption of distinct, even personalized, options for imaging and therapy of cancers. What will be important in the coming years is to strike a balance between the clear advantages of nano-radiopharmaceuticals in terms of radioisotope loading efficiency, tumor specificity, and therapeutic/diagnostic efficacy and the potential disadvantages of potentially unfavorable elimination kinetics and scale-up manufacturing/regulatory challenges. Again, this can only be achieved when a cadre of interdisciplinary cross-fertilized scientists embarks on advancing convergent science at the intersection of nanomedicine and nuclear medicine. Given current trends in research endeavors in this arena and early preclinical promise, we envision an optimistic outlook where novel agents can advance seamlessly from the bench to the bedside in the foreseeable future.

## Data Availability

Not applicable.

## References

[CR1] Abou DS, Thorek DLJ, Ramos NN, Pinkse MWH, Wolterbeek HT, Carlin SD, Beattie BJ, Lewis JS (2013). 89Zr-Labeled paramagnetic octreotide-liposomes for PET-MR imaging of cancer. Pharm Res.

[CR2] Akca O, Unak P, Medine EI, Sakarya S, Kilcar AY, Ichedef C, Bekis R, Timur S (2014). Radioiodine labeled CdSe/CdS quantum dots: lectin targeted dual probes. Radiochim Acta.

[CR3] Almutairi A, Rossin R, Shokeen M, Hagooly A, Ananth A, Capoccia B, Guillaudeu S, Abendschein D, Anderson CJ, Welch MJ (2009). Biodegradable dendritic positron-emitting nanoprobes for the noninvasive imaging of angiogenesis. Proc Natl Acad Sci USA.

[CR4] Anarjan FS (2019). Active targeting drug delivery nanocarriers: ligands. Nano Struct Nano Objects..

[CR5] Aranda-Lara L, Morales-Avila E, Luna-Gutiérrez MA, Olivé-Alvarez E, Isaac-Olivé K (2020). Radiolabeled liposomes and lipoproteins as lipidic nanoparticles for imaging and therapy. Chem Phys Lipids.

[CR6] Benjamin LE, Golijanin D, Itin A, Pode D, Keshet E (1999). Selective ablation of immature blood vessels in established human tumors follows vascular endothelial growth factor withdrawal. J Clin Invest.

[CR7] Black KCL, Akers WJ, Sudlow G, Xu B, Laforest R, Achilefu S (2014). Dual-radiolabeled nanoparticle SPECT probes for bioimaging. Nanoscale.

[CR8] Black KCL, Ibricevic A, Gunsten SP, Flores JA, Gustafson TP, Raymond JE, Samarajeewa S, Shrestha R, Felder SE, Cai T (2016). In vivo fate tracking of degradable nanoparticles for lung gene transfer using PET and ĉerenkov imaging. Biomaterials.

[CR9] Boersma YL, Plückthun A (2011). DARPins and other repeat protein scaffolds: advances in engineering and applications. Curr Opin Biotechnol.

[CR10] Boros E, Bowen AM, Josephson L, Vasdev N, Holland JP (2015). Chelate-free metal ion binding and heat-induced radiolabeling of iron oxide nanoparticles. Chem Sci.

[CR11] Boudjemeline M, Hopewell R, Rochon P-L, Jolly D, Hammami I, Villeneuve S, Kostikov A (2017). Highly efficient solid phase supported radiosynthesis of [11 C]PiB using TC18 cartridge as a “3-in-1” production entity. J Label Compd Radiopharm.

[CR12] Calvo-Alvarez E, Cren-Travaillé C, Crouzols A, Rotureau B (2018). A new chimeric triple reporter fusion protein as a tool for in vitro and in vivo multimodal imaging to monitor the development of african trypanosomes and leishmania parasites. Infect Genet Evol.

[CR13] Cao T, Zhou X, Zheng Y, Sun Y, Zhang J, Chen W, Zhang J-P, Zhou Z, Yang S-P, Zhang Y (2017). Chelator-free conjugation of 99mTc and Gd3+ to PEGylated nanographene oxide for dual-modality SPECT/MR imaging of lymph nodes. ACS Appl Mater Interfaces.

[CR14] Cędrowska E, Pruszyński M, Gawęda W, Żuk M, Krysiński P, Bruchertseifer F, Morgenstern A, Karageorgou M-A, Bouziotis P, Bilewicz A (2020). Trastuzumab conjugated superparamagnetic iron oxide nanoparticles labeled with 225Ac as a perspective tool for combined α-radioimmunotherapy and magnetic hyperthermia of HER2-positive breast cancer. Molecules.

[CR15] Chanda N, Kattumuri V, Shukla R, Zambre A, Katti K, Upendran A, Kulkarni RR, Kan P, Fent GM, Casteel SW (2010). Bombesin functionalized gold nanoparticles show in vitro and in vivo cancer receptor specificity. Proc Natl Acad Sci USA.

[CR16] Chen F, Ellison PA, Lewis CM, Hong H, Zhang Y, Shi S, Hernandez R, Meyerand ME, Barnhart TE, Cai W (2013). Chelator-free synthesis of a dual-modality PET/MRI agent. Angew Chem Int Ed Engl.

[CR17] Chen G, Roy I, Yang C, Prasad PN (2016). Nanochemistry and nanomedicine for nanoparticle-based diagnostics and therapy. Chem Rev.

[CR18] Chen S, Xu P, Li Y, Xue J, Han S, Ou W, Li L, Ni W (2016). Rapid seedless synthesis of gold nanoplates with microscaled edge length in a high yield and their application in SERS. Nanomicro Lett.

[CR19] Chen S, Yang K, Tuguntaev RG, Mozhi A, Zhang J, Wang PC, Liang X-J (2016). Targeting tumor microenvironment with PEG-based amphiphilic nanoparticles to overcome chemoresistance. Nanomedicine.

[CR20] Chen H, Zhang W, Zhu G, Xie J, Chen X (2017). Rethinking cancer nanotheranostics. Nat Rev Mater.

[CR21] Chen L, Chen J, Qiu S, Wen L, Wu Y, Hou Y, Wang Y, Zeng J, Feng Y, Li Z (2017). Biodegradable nanoagents with short biological half-life for SPECT/PAI/MRI multimodality imaging and PTT therapy of tumors. Small.

[CR22] Chen G, Zhang Y, Li C, Huang D, Wang Q, Wang Q (2018). Recent advances in tracking the transplanted stem cells using near-infrared fluorescent nanoprobes: turning from the first to the second near-infrared window. Adv Healthc Mater.

[CR23] Chen M, Guo Z, Chen Q, Wei J, Li J, Shi C, Xu D, Zhou D, Zhang X, Zheng N (2018). Pd nanosheets with their surface coordinated by radioactive iodide as a high-performance theranostic nanoagent for orthotopic hepatocellular carcinoma imaging and cancer therapy. Chem Sci.

[CR24] Chen M, Zhu W, Du J, Yang C, Han B, Zhou D, Huo L, Zhuang J (2021). 11C-acetate positron emission tomography is more precise than 18F-fluorodeoxyglucose positron emission tomography in evaluating tumor burden and predicting disease risk of multiple myeloma. Sci Rep.

[CR25] Cheng L, Wang C, Liu Z (2013). Upconversion nanoparticles and their composite nanostructures for biomedical imaging and cancer therapy. Nanoscale.

[CR26] Cheng L, Liu J, Gu X, Gong H, Shi X, Liu T, Wang C, Wang X, Liu G, Xing H (2014). PEGylated WS(2) nanosheets as a multifunctional theranostic agent for in vivo dual-modal CT/photoacoustic imaging guided photothermal therapy. Adv Mater.

[CR27] Cheng K, Kothapalli S-R, Liu H, Koh AL, Jokerst JV, Jiang H, Yang M, Li J, Levi J, Wu JC (2014). Construction and validation of nano gold tripods for molecular imaging of living subjects. J Am Chem Soc.

[CR28] Cheng L, Shen S, Shi S, Yi Y, Wang X, Song G, Yang K, Liu G, Barnhart TE, Cai W (2016). FeSe2-decorated Bi2Se3 nanosheets fabricated via cation exchange for chelator-free 64Cu-labeling and multimodal image-guided photothermal-radiation therapy. Adv Funct Mater.

[CR29] Cheng L, Shen S, Jiang D, Jin Q, Ellison PA, Ehlerding EB, Goel S, Song G, Huang P, Barnhart TE (2017). Chelator-free labeling of metal oxide nanostructures with zirconium-89 for positron emission tomography imaging. ACS Nano.

[CR30] Chouly C, Pouliquen D, Lucet I, Jeune JJ, Jallet P (1996). Development of superparamagnetic nanoparticles for MRI: effect of particle size, charge and surface nature on biodistribution. J Microencapsul.

[CR31] Cisneros BT, Law JJ, Matson ML, Azhdarinia A, Sevick-Muraca EM, Wilson LJ (2014). Stable confinement of positron emission tomography and magnetic resonance agents within carbon nanotubes for bimodal imaging. Nanomedicine (lond).

[CR32] Coenen HH, Mertens J, Mazière B, Bläuenstein P (2006). Radioionidation reactions for radiopharmaceuticals: compendium for effective synthesis strategies, ISBN 978–1–4020–4561–5.

[CR33] Coenen HH, Gee AD, Adam M, Antoni G, Cutler CS, Fujibayashi Y, Jeong JM, Mach RH, Mindt TL, Pike VW (2018). Open letter to journal editors on: International consensus radiochemistry nomenclature guidelines. Ann Nucl Med.

[CR34] Currie G, Wheat J, Davidson R, Kiat H (2011). Radionuclide production. Radiographer.

[CR35] da Silva WM, de Alves Andrade E, Silva RH, Cipreste MF, Andrade GF, Gastelois PL, de Almeida Macedo WA, de Sousa EMB (2020). Boron nitride nanotubes radiolabeled with 153Sm and 159Gd: potential application in nanomedicine. Appl Radiat Isot.

[CR36] Dash A, Chakravarty R (2019). Radionuclide generators: the prospect of availing PET radiotracers to meet current clinical needs and future research demands. Am J Nucl Med Mol Imaging.

[CR37] Dash A, Pillai MRA, Knapp FF (2015). Production of 177Lu for targeted radionuclide therapy: available options. Nucl Med Mol Imaging.

[CR38] De Simone M, Panetta D, Bramanti E, Giordano C, Salvatici MC, Gherardini L, Menciassi A, Burchielli S, Cinti C, Salvadori PA (2016). Magnetically driven nanoparticles: 18FDG-radiolabelling and positron emission tomography biodistribution study. Contrast Media Mol Imaging.

[CR39] Di Mauro PP, Gómez-Vallejo V, Baz Maldonado Z, Llop Roig J, Borrós S (2015). Novel 18F labeling strategy for polyester-based NPs for in vivo PET-CT imaging. Bioconjugate Chem.

[CR40] Di Pasqua AJ, Yuan H, Chung Y, Kim J-K, Huckle JE, Li C, Sadgrove M, Tran TH, Jay M, Lu X (2013). Neutron-activatable holmium-containing mesoporous silica nanoparticles as a potential radionuclide therapeutic agent for ovarian cancer. J Nucl Med.

[CR41] D’Huyvetter M, Aerts A, Xavier C, Vaneycken I, Devoogdt N, Gijs M, Impens N, Baatout S, Ponsard B, Muyldermans S (2012). Development of 177Lu-nanobodies for radioimmunotherapy of HER2-positive breast cancer: evaluation of different bifunctional chelators. Contrast Media Mol Imaging.

[CR42] Ding E-X, Wang J, Geng H-Z, Wang W-Y, Wang Y, Zhang Z-C, Luo Z-J, Yang H-J, Zou C-X, Kang J (2015). Y-junction carbon nanocoils: synthesis by chemical vapor deposition and formation mechanism. Sci Rep.

[CR43] Doane TL, Burda C (2012). The unique role of nanoparticles in nanomedicine: imaging, drug delivery and therapy. Chem Soc Rev.

[CR44] Dong K, Liu Z, Liu J, Huang S, Li Z, Yuan Q, Ren J, Qu X (2014). Biocompatible and high-performance amino acids-capped MnWO4 nanocasting as a novel non-lanthanide contrast agent for X-ray computed tomography and T(1)-weighted magnetic resonance imaging. Nanoscale.

[CR45] Duan Y, Liu B (2018). Recent advances of optical imaging in the second near-infrared window. Adv Mater.

[CR46] Dubey N, Shukla J, Hazari PP, Varshney R, Ganeshpurkar A, Mishra AK, Trivedi P, Bandopadhaya GP (2012). Preparation and biological evaluation of paclitaxel loaded biodegradable PCL/PEG nanoparticles for the treatment of human neuroendocrine pancreatic tumor in mice. Hell J Nucl Med.

[CR47] Duncan R (1999). Polymer conjugates for tumour targeting and intracytoplasmic delivery. The EPR effect as a common gateway?. Pharm Sci Technol Today.

[CR48] Ekinci M, Santos-Oliveira R, Ilem-Ozdemir D (2022). Biodistribution of 99mTc-PLA/PVA/atezolizumab nanoparticles for non-small cell lung cancer diagnosis. Eur J Pharm Biopharm.

[CR49] El-Marakby EM, Hathout RM, Taha I, Mansour S, Mortada ND (2017). A novel serum-stable liver targeted cytotoxic system using valerate-conjugated chitosan nanoparticles surface decorated with glycyrrhizin. Int J Pharm.

[CR50] European Directorate for the Quality of Medicines and HealthCare – EDQM (2023) Revised Guidance for Elaborating Monographs on Radiopharmaceutical Preparations: New Section on Validation of Methods. https://www.edqm.eu/en/-/revised-guidance-for-elaborating-monographs-on-radiopharmaceutical-preparations-new-section-on-validation-of-methods. Accessed 3 Feb 2023

[CR51] Fan Q, Cheng K, Hu X, Ma X, Zhang R, Yang M, Lu X, Xing L, Huang W, Gambhir SS (2014). Transferring biomarker into molecular probe: melanin nanoparticle as a naturally active platform for multimodality imaging. J Am Chem Soc.

[CR52] Fang J, Islam W, Maeda H (2020). Exploiting the dynamics of the EPR effect and strategies to improve the therapeutic effects of nanomedicines by using EPR effect enhancers. Adv Drug Deliv Rev.

[CR53] Farokhzad OC, Langer R (2009). Impact of nanotechnology on drug delivery. ACS Nano.

[CR54] Farzin L, Shamsipur M, Sheibani S (2016). Solid phase extraction of hemin from serum of breast cancer patients using an ionic liquid coated Fe3O4/Graphene oxide nanocomposite, and its quantitation by using FAAS. Microchim Acta.

[CR55] Farzin L, Sheibani S, Moassesi M, Shamsipur M (2018). An overview of nanoscale radionuclides and radiolabeled nanomaterials commonly used for nuclear molecular imaging and therapeutic functions. J Biomed Mater Res Part A.

[CR56] Fass L (2008). Imaging and cancer: a review. Mol Oncol.

[CR57] Fatouros PP, Corwin FD, Chen Z-J, Broaddus WC, Tatum JL, Kettenmann B, Ge Z, Gibson HW, Russ JL, Leonard AP (2006). In vitro and in vivo imaging studies of a new endohedral metallofullerene nanoparticle. Radiology.

[CR58] Fayez H, El-Motaleb MA, Selim AA (2020). Synergistic cytotoxicity of shikonin-silver nanoparticles as an opportunity for lung cancer. J Labell Comp Radiopharm.

[CR59] Fernandes RS, Silva JO, Seabra HA, Oliveira MS, Carregal VM, Vilela JMC, Andrade MS, Townsend DM, Colletti PM, Leite EA (2018). α- Tocopherol succinate loaded nano-structed lipid carriers improves antitumor activity of doxorubicin in breast cancer models in vivo. Biomed Pharmacother.

[CR60] Ferreira C, Ni D, Rosenkrans Z, Cai W (2019). Radionuclide-activated nanomaterials and their biomedical applications. Angew Chem.

[CR61] Ferro-Flores G, Ocampo-García BE, Santos-Cuevas CL, Morales-Avila E, Azorín-Vega E (2014). Multifunctional radiolabeled nanoparticles for targeted therapy. Curr Med Chem.

[CR62] Fleischmann M, Hendra PJ, McQuillan AJ (1974). Raman spectra of pyridine adsorbed at a silver electrode. Chem Phys Lett.

[CR63] Franck D, Nann H, Davi P, Schubiger PA, Ametamey SM (2009). Faster analysis of radiopharmaceuticals using ultra performance liquid chromatography (UPLC^®^) in combination with low volume radio flow cell. Appl Radiat Isot.

[CR64] Gao X, Cui Y, Levenson RM, Chung LWK, Nie S (2004). In vivo cancer targeting and imaging with semiconductor quantum dots. Nat Biotechnol.

[CR65] Gao H, Liu X, Tang W, Niu D, Zhou B, Zhang H, Liu W, Gu B, Zhou X, Zheng Y (2016). 99mTc-conjugated manganese-based mesoporous silica nanoparticles for SPECT, PH-responsive MRI and anti-cancer drug delivery. Nanoscale.

[CR66] Gao Z, Hou Y, Zeng J, Chen L, Liu C, Yang W, Gao M (2017). Tumor microenvironment-triggered aggregation of antiphagocytosis 99m Tc-labeled Fe3 O4 nanoprobes for enhanced tumor imaging in vivo. Adv Mater.

[CR67] Garg I, Nathan MA, Packard AT, Kwon ED, Larson NB, Lowe V, Davis BJ, Haloi R, Mahon ML, Goenka AH (2021). 11C-Choline positron emission tomography/computed tomography for detection of disease relapse in patients with history of biochemically recurrent prostate cancer and prostate-specific antigen ≤0.1 Ng/Ml. J Cancer Res Ther.

[CR68] Ge J, Zhang Q, Zeng J, Gu Z, Gao M (2020). Radiolabeling nanomaterials for multimodality imaging: new insights into nuclear medicine and cancer diagnosis. Biomaterials.

[CR69] Gee AP, Sumstad D, Stanson J, Watson P, Proctor J, Kadidlo D, Koch E, Sprague J, Wood D, Styers D (2008). A multicenter comparison study between the endosafe^®^ PTS™ rapid-release testing system and traditional methods for detecting endotoxin in cell-therapy products. Cytotherapy.

[CR70] Ghiassian S, Yu L, Gobbo P, Nazemi A, Romagnoli T, Luo W, Luyt LG, Workentin MS (2019). Nitrone-modified gold nanoparticles: synthesis, characterization, and their potential as 18F-labeled positron emission tomography probes via I-SPANC. ACS Omega.

[CR71] Goel S, England CG, Chen F, Cai W (2017). Positron emission tomography and nanotechnology: a dynamic duo for cancer theranostics. Adv Drug Deliv Rev.

[CR72] Guerrero S, Herance JR, Rojas S, Mena JF, Gispert JD, Acosta GA, Albericio F, Kogan MJ (2012). Synthesis and in vivo evaluation of the biodistribution of a 18F-labeled conjugate gold-nanoparticle-peptide with potential biomedical application. Bioconjugate Chem.

[CR73] Guo W, Sun X, Jacobson O, Yan X, Min K, Srivatsan A, Niu G, Kiesewetter DO, Chang J, Chen X (2015). Intrinsically radioactive [64Cu]CuInS/ZnS quantum dots for PET and optical imaging: improved radiochemical stability and controllable cerenkov luminescence. ACS Nano.

[CR74] Guo Z, Chen M, Peng C, Mo S, Shi C, Fu G, Wen X, Zhuang R, Su X, Liu T (2018). PH-sensitive radiolabeled and superfluorinated ultra-small palladium nanosheet as a high-performance multimodal platform for tumor theranostics. Biomaterials.

[CR75] Gupta S, Bansal R, Gupta S, Jindal N, Jindal A (2013). Nanocarriers and nanoparticles for skin care and dermatological treatments. Indian Dermatol Online J.

[CR76] Ha NS, Sadeghi S, Van Dam RM (2017). Recent progress toward microfluidic quality control testing of radiopharmaceuticals. Micromachines.

[CR77] Hamoudeh M, Kamleh MA, Diab R, Fessi H (2008). Radionuclides delivery systems for nuclear imaging and radiotherapy of cancer. Adv Drug Deliv Rev.

[CR78] Hansen AE, Petersen AL, Henriksen JR, Boerresen B, Rasmussen P, Elema DR, Rosenschöld PM, Kristensen AT, Kjær A, Andresen TL (2015). Positron emission tomography based elucidation of the enhanced permeability and retention effect in dogs with cancer using copper-64 liposomes. ACS Nano.

[CR79] He Z, Zhang Y, Feng N (2020). Cell membrane-coated nanosized active targeted drug delivery systems homing to tumor cells: a review. Mater Sci Eng C Mater Biol Appl.

[CR80] Hermanson GT (2013). Bioconjugate techniques, ISBN 978-0-12-382240-6.

[CR81] Higginbotham AL, Kosynkin DV, Sinitskii A, Sun Z, Tour JM (2010). Lower-defect graphene oxide nanoribbons from multiwalled carbon nanotubes. ACS Nano.

[CR82] Hiramatsu M, Hori M, Hiramatsu M, Hori M (2010). Physics of carbon nanowalls. Carbon nanowalls, ISBN 978–3–211–99717–8.

[CR83] Hong G, Antaris A, Dai H (2017). Near-infrared fluorophores for biomedical imaging. Nat Biomed Eng.

[CR84] Hori K, Zhang QH, Saito S, Tanda S, Li HC, Suzuki M (1993). Microvascular mechanisms of change in tumor blood flow due to angiotensin II, epinephrine, and methoxamine: a functional morphometric study. Cancer Res.

[CR85] Hu A, Wilson JJ (2022). Advancing chelation strategies for large metal ions for nuclear medicine applications. Acc Chem Res.

[CR86] Hu H, Li D, Liu S, Wang M, Moats R, Conti PS, Li Z (2014). Integrin Α2β1 targeted GdVO4: Eu ultrathin nanosheet for multimodal PET/MR imaging. Biomaterials.

[CR87] Hu H, Huang P, Weiss OJ, Yan X, Yue X, Zhang MG, Tang Y, Nie L, Ma Y, Niu G (2014). PET and NIR optical imaging using self-illuminating (64)Cu-doped chelator-free gold nanoclusters. Biomaterials.

[CR88] Hu Z, Chi C, Liu M, Guo H, Zhang Z, Zeng C, Ye J, Wang J, Tian J, Yang W (2017). Nanoparticle-mediated radiopharmaceutical-excited fluorescence molecular imaging allows precise image-guided tumor-removal surgery. Nanomedicine.

[CR89] Huang X, Peng X, Wang Y, Wang Y, Shin DM, El-Sayed MA, Nie S (2010). A reexamination of active and passive tumor targeting by using rod-shaped gold nanocrystals and covalently conjugated peptide ligands. ACS Nano.

[CR90] Islam R, Maeda H, Fang J (2022). Factors affecting the dynamics and heterogeneity of the EPR effect: pathophysiological and pathoanatomic features, drug formulations and physicochemical factors. Expert Opin Drug Deliv.

[CR91] Israel LL, Karimi F, Bianchessi S, Scanziani E, Passoni L, Matteoli M, Langström B, Lellouche J-P (2015). Surface metal cation doping of maghemite nanoparticles: modulation of MRI relaxivity features and chelator-free 68Ga-radiolabelling for dual MRI-PET imaging. Mater Res Express..

[CR92] Jabbour E, O’Brien S, Ravandi F, Kantarjian H (2015). Monoclonal antibodies in acute lymphoblastic leukemia. Blood.

[CR93] Jeanmaire DL, Van Duyne RP (1977). Surface Raman spectroelectrochemistry: part I. Heterocyclic, aromatic, and aliphatic amines adsorbed on the anodized silver electrode. J Electroanal Chem Interfacial Electrochem.

[CR94] Jeon J, Kang JA, Shim HE, Nam YR, Yoon S, Kim HR, Lee DE, Park SH (2015). Efficient method for iodine radioisotope labeling of cyclooctyne-containing molecules using strain-promoted copper-free click reaction. Bioorg Med Chem.

[CR95] Jewell EL, Huang JJ, Abu-Rustum NR, Gardner GJ, Brown CL, Sonoda Y, Barakat RR, Levine DA, Leitao MM (2014). Detection of sentinel lymph nodes in minimally invasive surgery using indocyanine green and near-infrared fluorescence imaging for uterine and cervical malignancies. Gynecol Oncol.

[CR96] Jiang C, Wu D, Haacke EM (2017). Ferritin-EGFP chimera as an endogenous dual-reporter for both fluorescence and magnetic resonance imaging in human glioma U251 cells. Tomography.

[CR97] Jiang X, Han Y, Zhang H, Liu H, Huang Q, Wang T, Sun Q, Li Z (2018). Cu–Fe–Se ternary nanosheet-based drug delivery carrier for multimodal imaging and combined chemo/photothermal therapy of cancer. ACS Appl Mater Interfaces.

[CR98] Jin Q, Zhu W, Jiang D, Zhang R, Kutyreff CJ, Engle JW, Huang P, Cai W, Liu Z, Cheng L (2017). Ultra-small iron-gallic acid coordination polymer nanoparticles for chelator-free labeling of 64Cu and multimodal imaging-guided photothermal therapy. Nanoscale.

[CR99] Jo SD, Ku SH, Won Y-Y, Kim SH, Kwon IC (2016). Targeted nanotheranostics for future personalized medicine: recent progress in cancer therapy. Theranostics.

[CR100] Kang KW, Song MG, Lee DS (2018). Organic nanomaterials: liposomes, albumin, dendrimer, polymeric nanoparticles. Radionanomedicine biological and medical physics biomedical engineering, ISBN 978-3-319-67719-4.

[CR101] Kasivisvanathan V, Rannikko AS, Borghi M, Panebianco V, Mynderse LA, Vaarala MH, Briganti A, Budäus L, Hellawell G, Hindley RG (2018). MRI-targeted or standard biopsy for prostate-cancer diagnosis. N Engl J Med.

[CR102] Kassis AI (2008). Therapeutic radionuclides: biophysical and radiobiologic principles. Semin Nucl Med.

[CR103] Kassis AI, Adelstein SJ (2005). Radiobiologic principles in radionuclide therapy. J Nucl Med.

[CR104] Katifelis H, Mukha I, Bouziotis P, Vityuk N, Tsoukalas C, Lazaris AC, Lyberopoulou A, Theodoropoulos GE, Efstathopoulos EP, Gazouli M (2020). Ag/Au bimetallic nanoparticles inhibit tumor growth and prevent metastasis in a mouse model. Int J Nanomedicine.

[CR105] Kato H, Huang X, Kadonaga Y, Katayama D, Ooe K, Shimoyama A, Kabayama K, Toyoshima A, Shinohara A, Hatazawa J (2021). Intratumoral administration of astatine-211-labeled gold nanoparticle for alpha therapy. J Nanobiotechnology.

[CR106] Keliher EJ, Ye Y-X, Wojtkiewicz GR, Aguirre AD, Tricot B, Senders ML, Groenen H, Fay F, Perez-Medina C, Calcagno C (2017). Polyglucose nanoparticles with renal elimination and macrophage avidity facilitate PET imaging in ischaemic heart disease. Nat Commun.

[CR107] Kennel SJ, Woodward JD, Rondinone AJ, Wall J, Huang Y, Mirzadeh S (2008). The fate of MAb-targeted Cd(125m)Te/ZnS nanoparticles in vivo. Nucl Med Biol.

[CR108] Kim D, Park S, Lee JH, Jeong YY, Jon S (2007). Antibiofouling polymer-coated gold nanoparticles as a contrast agent for in vivo X-ray computed tomography imaging. J Am Chem Soc.

[CR109] Kim S, Chae MK, Yim MS, Jeong IH, Cho J, Lee C, Ryu EK (2013). Hybrid PET/MR imaging of tumors using an oleanolic acid-conjugated nanoparticle. Biomaterials.

[CR110] Kim J-W, Porte Y, Ko KY, Kim H, Myoung J-M (2017). Micropatternable double-faced ZnO nanoflowers for flexible gas sensor. ACS Appl Mater Interfaces.

[CR111] Kircher MF, de la Zerda A, Jokerst JV, Zavaleta CL, Kempen PJ, Mittra E, Pitter K, Huang R, Campos C, Habte F (2012). A brain tumor molecular imaging strategy using a new triple-modality MRI-photoacoustic-raman nanoparticle. Nat Med.

[CR112] Kostiv U, Lobaz V, Kučka J, Švec P, Sedláček O, Hrubý M, Janoušková O, Francová P, Kolářová V, Šefc L (2017). A simple neridronate-based surface coating strategy for upconversion nanoparticles: highly colloidally stable 125 I-radiolabeled NaYF 4: Yb 3+ /Er 3+ @PEG nanoparticles for multimodal in vivo tissue imaging. Nanoscale.

[CR113] Králik M (2014). Adsorption, chemisorption, and catalysis. Chem Pap.

[CR114] Kreyling WG, Möller W, Holzwarth U, Hirn S, Wenk A, Schleh C, Schäffler M, Haberl N, Gibson N, Schittny JC (2018). Age-dependent rat lung deposition patterns of inhaled 20 nanometer gold nanoparticles and their quantitative biokinetics in adult rats. ACS Nano.

[CR115] Kumar K, Woolum K (2021). A novel reagent for radioiodine labeling of new chemical entities (NCEs) and biomolecules. Molecules.

[CR116] Kumar R, Roy I, Ohulchanskyy TY, Goswami LN, Bonoiu AC, Bergey EJ, Tramposch KM, Maitra A, Prasad PN (2008). Covalently dye-linked, surface-controlled, and bioconjugated organically modified silica nanoparticles as targeted probes for optical imaging. ACS Nano.

[CR117] Kumari P, Ghosh B, Biswas S (2016). Nanocarriers for cancer-targeted drug delivery. J Drug Target.

[CR118] Kunjachan S, Ehling J, Storm G, Kiessling F, Lammers T (2015). Noninvasive imaging of nanomedicines and nanotheranostics: principles, progress, and prospects. Chem Rev.

[CR119] Kuntić V, Brborić J, Vujić Z, Uskoković-Marković S (2016). Radioisotopes used as radiotracers for in vitro and in vivo diagnostics. Asian J Chem.

[CR120] Lamb J, Holland JP (2018). Advanced methods for radiolabeling multimodality nanomedicines for SPECT/MRI and PET/MRI. J Nucl Med.

[CR121] Lee N, Choi SH, Hyeon T (2013). Nano-sized CT contrast agents. Adv Mater.

[CR122] Lee S, Hong J, Koo JH, Lee H, Lee S, Choi T, Jung H, Koo B, Park J, Kim H (2013). Synthesis of few-layered graphene nanoballs with copper cores using solid carbon source. ACS Appl Mater Interfaces.

[CR123] Lemaître TA, Burgoyne AR, Ooms M, Parac-Vogt TN, Cardinaels T (2022). Inorganic radiolabeled nanomaterials in cancer therapy: a review. ACS Appl Nano Mater.

[CR124] Leu AJ, Berk DA, Lymboussaki A, Alitalo K, Jain RK (2000). Absence of functional lymphatics within a murine sarcoma: a molecular and functional evaluation. Cancer Res.

[CR125] Li J, Pu K (2019). Development of organic semiconducting materials for deep-tissue optical imaging, phototherapy and photoactivation. Chem Soc Rev.

[CR126] Li Y, Lin T-Y, Luo Y, Liu Q, Xiao W, Guo W, Lac D, Zhang H, Feng C, Wachsmann-Hogiu S (2014). A smart and versatile theranostic nanomedicine platform based on nanoporphyrin. Nat Commun.

[CR127] Li J, Hu Y, Yang J, Wei P, Sun W, Shen M, Zhang G, Shi X (2015). Hyaluronic acid-modified Fe3O4@Au core/shell nanostars for multimodal imaging and photothermal therapy of tumors. Biomaterials.

[CR128] Li C-H, Kuo T-R, Su H-J, Lai W-Y, Yang P-C, Chen J-S, Wang D-Y, Wu Y-C, Chen C-C (2015). Fluorescence-guided probes of aptamer-targeted gold nanoparticles with computed tomography imaging accesses for in vivo tumor resection. Sci Rep.

[CR129] Li X, Xiong Z, Xu X, Luo Y, Peng C, Shen M, Shi X (2016). 99mTc-labeled multifunctional low-generation dendrimer-entrapped gold nanoparticles for targeted SPECT/CT dual-mode imaging of tumors. ACS Appl Mater Interfaces.

[CR130] Li Y, Li X, Xue Z, Jiang M, Zeng S, Hao J (2017). M2+ doping induced simultaneous phase/size control and remarkable enhanced upconversion luminescence of NaLnF4 probes for optical-guided tiny tumor diagnosis. Adv Healthc Mater.

[CR131] Li Y, Li X, Xue Z, Jiang M, Zeng S, Hao J (2018). Second near-infrared emissive lanthanide complex for fast renal-clearable in vivo optical bioimaging and tiny tumor detection. Biomaterials.

[CR132] Li Y, Zeng S, Hao J (2019). Non-invasive optical guided tumor metastasis/vessel imaging by using lanthanide nanoprobe with enhanced down-shifting emission beyond 1500 nm. ACS Nano.

[CR133] Liberale G, Bohlok A, Bormans A, Bouazza F, Galdon MG, El Nakadi I, Bourgeois P, Donckier V (2018). Indocyanine green fluorescence imaging for sentinel lymph node detection in colorectal cancer: a systematic review. Eur J Surg Oncol.

[CR134] Lim E-K, Kim T, Paik S, Haam S, Huh Y-M, Lee K (2015). Nanomaterials for theranostics: recent advances and future challenges. Chem Rev.

[CR135] Lin X, Xie J, Niu G, Zhang F, Gao H, Yang M, Quan Q, Aronova MA, Zhang G, Lee S (2011). Chimeric ferritin nanocages for multiple function loading and multimodal imaging. Nano Lett.

[CR136] Lin W, Ma G, Kampf N, Yuan Z, Chen S (2016). Development of long-circulating Zwitterionic cross-linked micelles for active-targeted drug delivery. Biomacromol.

[CR137] Lipi F, Chen S, Chakravarthy M, Rakesh S, Veedu RN (2016). In vitro evolution of chemically-modified nucleic acid aptamers: pros and cons, and comprehensive selection strategies. RNA Biol.

[CR138] Liu Z, Ma R, Ebina Y, Takada K, Sasaki T (2007). Synthesis and delamination of layered manganese oxide nanobelts. Chem Mater.

[CR139] Liu Y, Ibricevic A, Cohen JA, Cohen JL, Gunsten SP, Fréchet JMJ, Walter MJ, Welch MJ, Brody SL (2009). Impact of hydrogel nanoparticle size and functionalization on in vivo behavior for lung imaging and therapeutics. Mol Pharm.

[CR140] Liu S, Jia B, Qiao R, Yang Z, Yu Z, Liu Z, Liu K, Shi J, Ouyang H, Wang F (2009). A novel type of dual-modality molecular probe for MR and nuclear imaging of tumor: preparation, characterization and in vivo application. Mol Pharm.

[CR141] Liu Q, Sun Y, Li C, Zhou J, Li C, Yang T, Zhang X, Yi T, Wu D, Li F (2011). 18F-labeled magnetic-upconversion nanophosphors via rare-earth cation-assisted ligand assembly. ACS Nano.

[CR142] Liu TW, MacDonald TD, Shi J, Wilson BC, Zheng G (2012). Intrinsically copper-64-labeled organic nanoparticles as radiotracers. Angew Chem Int Ed Engl.

[CR143] Liu Z, Pu F, Liu J, Jiang L, Yuan Q, Li Z, Ren J, Qu X (2013). PEGylated hybrid ytterbia nanoparticles as high-performance diagnostic probes for in vivo magnetic resonance and X-ray computed tomography imaging with low systemic toxicity. Nanoscale.

[CR144] Liu C, Gao Z, Zeng J, Hou Y, Fang F, Li Y, Qiao R, Shen L, Lei H, Yang W (2013). Magnetic/upconversion fluorescent NaGdF4:Yb, Er nanoparticle-based dual-modal molecular probes for imaging tiny tumors in vivo. ACS Nano.

[CR145] Liu Y, Sun Y, Cao C, Yang Y, Wu Y, Ju D, Li F (2014). Long-term biodistribution in vivo and toxicity of radioactive/magnetic hydroxyapatite nanorods. Biomaterials.

[CR146] Liu T, Shi S, Liang C, Shen S, Cheng L, Wang C, Song X, Goel S, Barnhart TE, Cai W (2015). Iron oxide decorated MoS2 nanosheets with double PEGylation for chelator-free radiolabeling and multimodal imaging guided photothermal therapy. ACS Nano.

[CR147] Liu Y, Bhattarai P, Dai Z, Chen X (2019). Photothermal therapy and photoacoustic imaging via nanotheranostics in fighting cancer. Chem Soc Rev.

[CR148] Liu Z, Parida S, Prasad R, Pandey R, Sharma D, Barman I (2021). Vibrational spectroscopy for decoding cancer microbiota interactions: current evidence and future perspective. Semin Cancer Biol.

[CR149] Löfblom J, Feldwisch J, Tolmachev V, Carlsson J, Ståhl S, Frejd FY (2010). Affibody molecules: engineered proteins for therapeutic, diagnostic and biotechnological applications. FEBS Lett.

[CR150] Luderer MJ, de la Puente P, Azab AK (2015). Advancements in tumor targeting strategies for boron neutron capture therapy. Pharm Res.

[CR151] Luebke AL, Wilary DM, Mahoney DW, Hung JC (2000). Evaluation of an alternative radiochemical purity testing method for technetium-99m-sestamibi. J Nucl Med Technol.

[CR152] Lumen D, Näkki S, Imlimthan S, Lambidis E, Sarparanta M, Xu W, Lehto V-P, Airaksinen AJ (2019). Site-specific 111In-radiolabeling of dual-PEGylated porous silicon nanoparticles and their in vivo evaluation in murine 4T1 breast cancer model. Pharmaceutics.

[CR153] Madru R, Kjellman P, Olsson F, Wingårdh K, Ingvar C, Ståhlberg F, Olsrud J, Lätt J, Fredriksson S, Knutsson L (2012). 99mTc-labeled superparamagnetic iron oxide nanoparticles for multimodality SPECT/MRI of sentinel lymph nodes. J Nucl Med.

[CR154] Maeda H, Wu J, Sawa T, Matsumura Y, Hori K (2000). Tumor vascular permeability and the EPR effect in macromolecular therapeutics: a review. J Control Release.

[CR155] Mahato R (2017). Nanoemulsion as targeted drug delivery system for cancer therapeutics. J Pharm Sci Pharmacol.

[CR156] Man F, Gawne PJ, de Rosales RT (2019). Nuclear imaging of liposomal drug delivery systems: a critical review of radiolabelling methods and applications in nanomedicine. Adv Drug Deliv Rev.

[CR157] Marín A, Martín M, Liñán O, Alvarenga F, López M, Fernández L, Büchser D, Cerezo L (2014). Bystander effects and radiotherapy. Rep Pract Oncol Radiother.

[CR158] Masood F (2016). Polymeric nanoparticles for targeted drug delivery system for cancer therapy. Mater Sci Eng C Mater Biol Appl.

[CR159] Matesic L, Kallinen A, Greguric I, Pascali G (2017). Dose-on-demand production of diverse 18F-radiotracers for preclinical applications using a continuous flow microfluidic system. Nucl Med Biol.

[CR160] Matsumura Y, Maeda H (1986). A new concept for macromolecular therapeutics in cancer chemotherapy: mechanism of tumoritropic accumulation of proteins and the antitumor agent smancs. Cancer Res.

[CR161] Mattoli MV, Trevisi G, Scolozzi V, Capotosti A, Cocciolillo F, Marini I, Mare V, Indovina L, Caulo M, Saponiero A (2021). Dynamic 11C-methionine PET-CT: prognostic factors for disease progression and survival in patients with suspected glioma recurrence. Cancers (basel).

[CR162] Meyer J-P, Adumeau P, Lewis JS, Zeglis BM (2016). Click chemistry and radiochemistry: the first 10 years. Bioconjugate Chem.

[CR163] Miao Q, Pu K (2018). Organic semiconducting agents for deep-tissue molecular imaging: second near-infrared fluorescence, self-luminescence, and photoacoustics. Adv Mater.

[CR164] Michalet X, Pinaud FF, Bentolila LA, Tsay JM, Doose S, Li JJ, Sundaresan G, Wu AM, Gambhir SS, Weiss S (2005). Quantum dots for live cells, in vivo imaging, and diagnostics. Science.

[CR165] Min Y, Caster JM, Eblan MJ, Wang AZ (2015). Clinical translation of nanomedicine. Chem Rev.

[CR166] Miranda SE, de Alcântara Lemos J, Fernandes RS, de Oliveira Silva J, Ottoni FM, Townsend DM, Rubello D, Alves RJ, Cassali GD, Ferreira LA (2021). Enhanced antitumor efficacy of lapachol-loaded nanoemulsion in breast cancer tumor model. Biomed Pharmacother.

[CR167] Misri R, Meier D, Yung AC, Kozlowski P, Häfeli UO (2012). Development and evaluation of a dual-modality (MRI/SPECT) molecular imaging bioprobe. Nanomedicine.

[CR168] Mitchell GS, Gill RK, Boucher DL, Li C, Cherry SR (2011). In vivo cerenkov luminescence imaging: a new tool for molecular imaging. Philos Trans A Math Phys Eng Sci.

[CR169] Mitra A, Nan A, Line BR, Ghandehari H (2006). Nanocarriers for nuclear imaging and radiotherapy of cancer. Curr Pharm Des.

[CR170] Molavipordanjani S, Tolmachev V, Hosseinimehr SJ (2019). Basic and practical concepts of radiopharmaceutical purification methods. Drug Discov Today.

[CR171] Mueller D, Breeman WAP, Klette I, Gottschaldt M, Odparlik A, Baehre M, Tworowska I, Schultz MK (2016). Radiolabeling of DOTA-like conjugated peptides with generator-produced (68)Ga and using NaCl-based cationic elution method. Nat Protoc.

[CR172] Munaweera I, Shi Y, Koneru B, Saez R, Aliev A, Di Pasqua AJ, Balkus KJ (2015). Chemoradiotherapeutic magnetic nanoparticles for targeted treatment of nonsmall cell lung cancer. Mol Pharm.

[CR173] Mura S, Couvreur P (2012). Nanotheranostics for personalized medicine. Adv Drug Deliv Rev.

[CR174] Muralidharan P, Malapit M, Mallory E, Hayes D, Mansour HM (2015). Inhalable nanoparticulate powders for respiratory delivery. Nanomedicine.

[CR175] Nag OK, Delehanty JB (2019). Active cellular and subcellular targeting of nanoparticles for drug delivery. Pharmaceutics.

[CR176] Nagarajan S, Belaid H, Pochat-Bohatier C, Teyssier C, Iatsunskyi I, Coy E, Balme S, Cornu D, Miele P, Kalkura NS (2017). Design of boron nitride/gelatin electrospun nanofibers for bone tissue engineering. ACS Appl Mater Interfaces.

[CR177] Nahrendorf M, Keliher E, Marinelli B, Waterman P, Feruglio PF, Fexon L, Pivovarov M, Swirski FK, Pittet MJ, Vinegoni C (2010). Hybrid PET-optical imaging using targeted probes. Med Sci.

[CR178] Nawaz S, Mullen GED, Blower PJ, Ballinger JR (2017). A 99mTc-labelled ScFv antibody fragment that binds to prostate-specific membrane antigen. Nucl Med Commun.

[CR179] Nayak D, Lahiri S (1999). Application of radioisotopes in the field of nuclear medicine. J Radioanal Nucl Chem.

[CR180] Nie S, Xing Y, Kim GJ, Simons JW (2007). Nanotechnology applications in cancer. Annu Rev Biomed Eng.

[CR181] Nijsen F, Rook D, Brandt C, Meijer R, Dullens H, Zonnenberg B, de Klerk J, van Rijk P, Hennink W, van het Schip F (2001). Targeting of liver tumour in rats by selective delivery of Holmium-166 loaded microspheres: a biodistribution study. Eur J Nucl Med.

[CR182] Nolte T, Gross-Weege N, Schulz V (2020). (Hybrid) SPECT and PET technologies. Recent Results Cancer Res.

[CR183] Ory D, Van den Brande J, de Groot T, Serdons K, Bex M, Declercq L, Cleeren F, Ooms M, Van Laere K, Verbruggen A (2015). Retention of [18F]fluoride on reversed phase HPLC columns. J Pharm Biomed Anal.

[CR184] Özyüncü SY, Teksöz S, Içhedef Ç, Medinel EI, Avci ÇB, Gündüz C, Ünak P (2016). Radiolabeled D-penicillamine magnetic nanocarriers for targeted purposes. J Nanosci Nanotechnol.

[CR185] Park J-A, Kim JY (2013). Recent advances in radiopharmaceutical application of matched-pair radiometals. Curr Top Med Chem.

[CR186] Park J-S, Kim I-K, Han S, Park I, Kim C, Bae J, Oh SJ, Lee S, Kim JH, Woo D-C (2016). Normalization of tumor vessels by Tie2 activation and Ang2 inhibition enhances drug delivery and produces a favorable tumor microenvironment. Cancer Cell.

[CR187] Peach K, Wilson P, Jones B (2011). Accelerator science in medical physics. Br J Radiol.

[CR188] Pelaz B, Alexiou C, Alvarez-Puebla RA, Alves F, Andrews AM, Ashraf S, Balogh LP, Ballerini L, Bestetti A, Brendel C (2017). Diverse applications of nanomedicine. ACS Nano.

[CR189] Pellico J, Ruiz-Cabello J, Saiz-Alía M, Del Rosario G, Caja S, Montoya M, Fernández de Manuel L, Morales MP, Gutiérrez L, Galiana B (2016). Fast synthesis and bioconjugation Of (68) Ga core-doped extremely small iron oxide nanoparticles for PET/MR imaging. Contrast Media Mol Imaging.

[CR190] Peltek OO, Muslimov AR, Zyuzin MV, Timin AS (2019). Current outlook on radionuclide delivery systems: from design consideration to translation into clinics. J Nanobiotechnology.

[CR191] Peng J, Sun Y, Zhao L, Wu Y, Feng W, Gao Y, Li F (2013). Polyphosphoric acid capping radioactive/upconverting NaLuF4:Yb, Tm,153Sm nanoparticles for blood pool imaging in vivo. Biomaterials.

[CR192] Pérez-Campaña C, Gómez-Vallejo V, Martin A, San Sebastián E, Moya SE, Reese T, Ziolo RF, Llop J (2012). Tracing nanoparticles in vivo: a new general synthesis of positron emitting metal oxide nanoparticles by proton beam activation. Analyst.

[CR193] Pérez-Campaña C, Gómez-Vallejo V, Puigivila M, Martín A, Calvo-Fernández T, Moya SE, Ziolo RF, Reese T, Llop J (2013). Biodistribution of different sized nanoparticles assessed by positron emission tomography: a general strategy for direct activation of metal oxide particles. ACS Nano.

[CR194] Pérez-Medina C, Abdel-Atti D, Zhang Y, Longo VA, Irwin CP, Binderup T, Ruiz-Cabello J, Fayad ZA, Lewis JS, Mulder WJM (2014). A modular labeling strategy for in vivo PET and near-infrared fluorescence imaging of nanoparticle tumor targeting. J Nucl Med.

[CR195] Pérez-Medina C, Teunissen AJP, Kluza E, Mulder WJ, van der Meel R (2020). Nuclear imaging approaches facilitating nanomedicine translation. Adv Drug Deliv Rev.

[CR196] Polyak A, Ross TL (2018). Nanoparticles for SPECT and PET imaging: towards personalized medicine and theranostics. Curr Med Chem.

[CR197] Poty S, Francesconi LC, McDevitt MR, Morris MJ, Lewis JS (2018). α-emitters for radiotherapy: from basic radiochemistry to clinical studies-part 1. J Nucl Med.

[CR198] Pouliquen D, Perdrisot R, Ermias A, Akoka S, Jallet P, Le Jeune JJ (1989). Superparamagnetic iron oxide nanoparticles as a liver MRI contrast agent: contribution of microencapsulation to improved biodistribution. Magn Reson Imaging.

[CR199] Prasad PN (2012). Introduction to nanomedicine and nanobioengineering; Wiley series in biomedical engineering and multidisciplinary integrated systems.

[CR200] Price EW, Orvig C (2014). Matching chelators to radiometals for radiopharmaceuticals. Chem Soc Rev.

[CR202] Qaim SM (2001). Therapeutic radionuclides and nuclear data. Radiochim Acta.

[CR203] Qian F, Lan PC, Freyman MC, Chen W, Kou T, Olson TY, Zhu C, Worsley MA, Duoss EB, Spadaccini CM (2017). Ultralight conductive silver nanowire aerogels. Nano Lett.

[CR204] Qiao R, Liu C, Liu M, Hu H, Liu C, Hou Y, Wu K, Lin Y, Liang J, Gao M (2015). Ultrasensitive in vivo detection of primary gastric tumor and lymphatic metastasis using upconversion nanoparticles. ACS Nano.

[CR205] Qiu S, Zeng J, Hou Y, Chen L, Ge J, Wen L, Liu C, Zhang Y, Zhu R, Gao M (2018). Detection of lymph node metastasis with near-infrared upconversion luminescent nanoprobes. Nanoscale.

[CR206] Ramogida CF, Orvig C (2013). Tumour targeting with radiometals for diagnosis and therapy. Chem Commun.

[CR207] Ranjbar Bahadori S, Mulgaonkar A, Hart R, Wu CY, Zhang D, Pillai A, Hao Y, Sun X (2021). Radiolabeling strategies and pharmacokinetic studies for metal based nanotheranostics. Wiley Interdiscip Rev Nanomed Nanobiotechnol..

[CR208] Reibel AT, Müller SS, Pektor S, Bausbacher N, Miederer M, Frey H, Rösch F (2015). Fate of linear and branched polyether-lipids in vivo in comparison to their liposomal formulations by 18F-radiolabeling and positron emission tomography. Biomacromol.

[CR209] Rhim W-K, Kim M, Hartman KL, Kang KW, Nam J-M (2015). Radionuclide-labeled nanostructures for in vivo imaging of cancer. Nano Converg.

[CR210] Roberts DW, Valdés PA, Harris BT, Fontaine KM, Hartov A, Fan X, Ji S, Lollis SS, Pogue BW, Leblond F (2011). Coregistered fluorescence-enhanced tumor resection of malignant glioma: relationships between δ-aminolevulinic acid-induced protoporphyrin IX fluorescence, magnetic resonance imaging enhancement, and neuropathological parameters. Clinical article. J Neurosurg.

[CR211] Roldan Cuenya B, Croy JR, Mostafa S, Behafarid F, Li L, Zhang Z, Yang JC, Wang Q, Frenkel AI (2010). Solving the structure of size-selected Pt nanocatalysts synthesized by inverse micelle encapsulation. J Am Chem Soc.

[CR212] Saha GB, Saha GB (2018). Nuclear pharmacy. Fundamentals of nuclear pharmacy, ISBN 978–3–319–57580–3.

[CR213] Sakr TM, Khowessah OM, Motaleb MA, Abd El-Bary A, El-Kolaly MT, Swidan MM (2018). I-131 doping of silver nanoparticles platform for tumor theranosis guided drug delivery. Eur J Pharm Sci.

[CR214] Selvan ST, Tan TT, Ying JY (2005). Robust, non-cytotoxic, silica-coated CdSe quantum dots with efficient photoluminescence. Adv Mater.

[CR215] Serdons K, Verbruggen A, Bormans G (2008). The presence of ethanol in radiopharmaceutical injections. J Nucl Med.

[CR216] Shaffer TM, Wall MA, Harmsen S, Longo VA, Drain CM, Kircher MF, Grimm J (2015). Silica nanoparticles as substrates for chelator-free labeling of oxophilic radioisotopes. Nano Lett.

[CR217] Shaffer TM, Harmsen S, Khwaja E, Kircher MF, Drain CM, Grimm J (2016). Stable radiolabeling of sulfur-functionalized silica nanoparticles with copper-64. Nano Lett.

[CR218] Shamsipur M, Farzin L, Tabrizi MA, Shanehsaz M (2016). CdTe amplification nanoplatforms capped with thioglycolic acid for electrochemical aptasensing of ultra-traces of ATP. Mater Sci Eng C Mater Biol Appl.

[CR219] Shamsipur M, Emami M, Farzin L, Saber R (2018). A sandwich-type electrochemical immunosensor based on in situ silver deposition for determination of serum level of HER2 in breast cancer patients. Biosens Bioelectron.

[CR220] Shao Y, Cherry SR, Farahani K, Meadors K, Siegel S, Silverman RW, Marsden PK (1997). Simultaneous PET and MR imaging. Phys Med Biol.

[CR221] Shukla J, Vatsa R, Garg N, Bhusari P, Watts A, Mittal BR (2013). Quality control of positron emission tomography radiopharmaceuticals: an institutional experience. Indian J Nucl Med.

[CR222] Sinha N, Cifter G, Sajo E, Kumar R, Sridhar S, Nguyen PL, Cormack RA, Makrigiorgos GM, Ngwa W (2015). Brachytherapy application with in situ dose painting administered by gold nanoparticle eluters. Int J Radiat Oncol Biol Phys.

[CR223] Smith BR, Gambhir SS (2017). Nanomaterials for in vivo imaging. Chem Rev.

[CR224] Sneddon D, Cornelissen B (2021). Emerging chelators for nuclear imaging. Curr Opin Chem Biol.

[CR225] Song G, Cheng L, Chao Y, Yang K, Liu Z (2017). Emerging nanotechnology and advanced materials for cancer radiation therapy. Adv Mater.

[CR226] Spinelli AE, Ferdeghini M, Cavedon C, Zivelonghi E, Calandrino R, Fenzi A, Sbarbati A, Boschi F (2013). First human cerenkography. J Biomed Opt.

[CR227] Stéen EJL, Jørgensen JT, Johann K, Nørregaard K, Sohr B, Svatunek D, Birke A, Shalgunov V, Edem PE, Rossin R (2020). Trans-cyclooctene-functionalized peptobrushes with improved reaction kinetics of the tetrazine ligation for pretargeted nuclear imaging. ACS Nano.

[CR228] Steinbrueck A, Sedgwick AC, Brewster JT, Yan K-C, Shang Y, Knoll DM, Vargas-Zúñiga GI, He X-P, Tian H, Sessler JL (2020). Transition metal chelators, pro-chelators, and ionophores as small molecule cancer chemotherapeutic agents. Chem Soc Rev.

[CR229] Sun M, Hoffman D, Sundaresan G, Yang L, Lamichhane N, Zweit J (2012). Synthesis and characterization of intrinsically radiolabeled quantum dots for bimodal detection. Am J Nucl Med Mol Imaging.

[CR230] Sun Y, Zhu X, Peng J, Li F (2013). Core-Shell lanthanide upconversion nanophosphors as four-modal probes for tumor angiogenesis imaging. ACS Nano.

[CR231] Sun X, Huang X, Guo J, Zhu W, Ding Y, Niu G, Wang A, Kiesewetter DO, Wang ZL, Sun S (2014). Self-illuminating 64Cu-doped CdSe/ZnS nanocrystals for in vivo tumor imaging. J Am Chem Soc.

[CR232] Sun M, Sundaresan G, Jose P, Yang L, Hoffman D, Lamichhane N, Zweit J (2014). Highly stable intrinsically radiolabeled indium-111 quantum dots with multidentate zwitterionic surface coating: dual modality tool for biological imaging. J Mater Chem B.

[CR233] Sun X, Cai W, Chen X (2015). Positron emission tomography imaging using radiolabeled inorganic nanomaterials. Acc Chem Res.

[CR234] Sun Z, Cheng K, Wu F, Liu H, Ma X, Su X, Liu Y, Xia L, Cheng Z (2016). Robust surface coating for a fast, facile fluorine-18 labeling of iron oxide nanoparticles for PET/MR dual-modality imaging. Nanoscale.

[CR235] Sun H, Zhang B, Jiang X, Liu H, Deng S, Li Z, Shi H (2019). Radiolabeled ultra-small Fe3O4 nanoprobes for tumor-targeted multimodal imaging. Nanomedicine (lond).

[CR236] Suzuki M, Hori K, Abe I, Saito S, Sato H (1981). A new approach to cancer chemotherapy: selective enhancement of tumor blood flow with angiotensin II. J Natl Cancer Inst.

[CR237] Tabrizi M, Shamsipur M, Farzin L, Molaabasi F (2015). Highly sensitive label free electrochemical detection of VGEF165 tumor marker based on “signal off” and “signal on” strategies using an anti-VEGF165 aptamer immobilized BSA-gold nanoclusters/ionic liquid/glassy carbon electrode. Biosens Bioelectron.

[CR238] Tang L, Yang X, Dobrucki LW, Chaudhury I, Yin Q, Yao C, Lezmi S, Helferich WG, Fan TM, Cheng J (2012). Aptamer-functionalized, ultra-small, monodisperse silica nanoconjugates for targeted dual-modal imaging of lymph nodes with metastatic tumors. Angew Chem.

[CR239] Tang J, Baxter S, Menon A, Alaarg A, Sanchez-Gaytan BL, Fay F, Zhao Y, Ouimet M, Braza MS, Longo VA (2016). Immune cell screening of a nanoparticle library improves atherosclerosis therapy. Proc Natl Acad Sci USA.

[CR240] Tang T, Wei Y, Yang Q, Yang Y, Sailor MJ, Pang H-B (2019). Rapid chelator-free radiolabeling of quantum dots for in vivo imaging. Nanoscale.

[CR241] Thorek DLJ, Riedl CC, Grimm J (2014). Clinical cerenkov luminescence imaging of 18F-FDG. J Nucl Med.

[CR242] Tian R, Zhao S, Liu G, Chen H, Ma L, You H, Liu C, Wang Z (2019). Construction of lanthanide-doped upconversion nanoparticle-Uelx Europaeus agglutinin-I bioconjugates with brightness red emission for ultrasensitive in vivo imaging of colorectal tumor. Biomaterials.

[CR243] Ting G, Chang CH, Wang HE, Lee TW (2010). Nanotargeted radionuclides for cancer nuclear imaging and internal radiotherapy. J Biomed Biotechnol.

[CR244] Torchilin V (2011). Tumor delivery of macromolecular drugs based on the EPR effect. Adv Drug Deliv Rev.

[CR245] Tsai W-TK, Wu AM (2018). Aligning physics and physiology: engineering antibodies for radionuclide delivery. J Labelled Comp Radiopharm.

[CR246] Tsentalovich DE, Headrick RJ, Mirri F, Hao J, Behabtu N, Young CC, Pasquali M (2017). Influence of carbon nanotube characteristics on macroscopic fiber properties. ACS Appl Mater Interfaces.

[CR247] Ulbrich K, Holá K, Šubr V, Bakandritsos A, Tuček J, Zbořil R (2016). Targeted drug delivery with polymers and magnetic nanoparticles: covalent and noncovalent approaches, release control, and clinical studies. Chem Rev.

[CR248] Valerio M, Donaldson I, Emberton M, Ehdaie B, Hadaschik BA, Marks LS, Mozer P, Rastinehad AR, Ahmed HU (2015). Detection of clinically significant prostate cancer using magnetic resonance imaging-ultrasound fusion targeted biopsy: a systematic review. Eur Urol.

[CR249] Vallières M, Freeman CR, Skamene SR, El Naqa I (2015). A radiomics model from joint FDG-PET and MRI texture features for the prediction of lung metastases in soft-tissue sarcomas of the extremities. Phys Med Biol.

[CR250] Velikyan I (2018). Prospective of 68Ga radionuclide contribution to the development of imaging agents for infection and inflammation. Contrast Media Mol Imaging.

[CR251] Volkert WA, Goeckeler WF, Ehrhardt GJ, Ketring AR (1991). Therapeutic radionuclides: production and decay property considerations. J Nucl Med.

[CR252] Wagener K, Worm M, Pektor S, Schinnerer M, Thiermann R, Miederer M, Frey H, Rösch F (2018). Comparison of linear and hyperbranched polyether lipids for liposome shielding by 18F-radiolabeling and positron emission tomography. Biomacromol.

[CR253] Wagner CC, Langer O (2011). Approaches using molecular imaging technology—use of PET in clinical microdose studies. Adv Drug Deliv Rev.

[CR254] Wall MA, Shaffer TM, Harmsen S, Tschaharganeh D-F, Huang C-H, Lowe SW, Drain CM, Kircher MF (2017). Chelator-free radiolabeling of SERRS nanoparticles for whole-body PET and intraoperative raman imaging. Theranostics.

[CR255] Wang F, Liu X (2009). Recent advances in the chemistry of lanthanide-doped upconversion nanocrystals. Chem Soc Rev.

[CR256] Wang Y, Liu Y, Luehmann H, Xia X, Wan D, Cutler C, Xia Y (2013). Radioluminescent gold nanocages with controlled radioactivity for real-time in vivo imaging. Nano Lett.

[CR257] Wang Z, Huang P, Jacobson O, Wang Z, Liu Y, Lin L, Lin J, Lu N, Zhang H, Tian R (2016). Biomineralization-inspired synthesis of copper sulfide-ferritin nanocages as cancer theranostics. ACS Nano.

[CR258] Wang Y, Wu Y, Liu Y, Shen J, Lv L, Li L, Yang L, Zeng J, Wang Y, Zhang LW (2016). BSA-mediated synthesis of bismuth sulfide nanotheranostic agents for tumor multimodal imaging and thermoradiotherapy. Adv Funct Mater.

[CR259] Wang J, Chao PH, Hanet S, van Dam RM (2017). Performing multi-step chemical reactions in microliter-sized droplets by leveraging a simple passive transport mechanism. Lab Chip.

[CR260] Wang H, Mu X, Yang J, Liang Y, Zhang X-D, Ming D (2019). Brain imaging with near-infrared fluorophores. Coord Chem Rev.

[CR261] Wang Z, Ye M, Ma D, Shen J, Fang F (2022). Engineering of 177Lu-labeled gold encapsulated into dendrimeric nanomaterials for the treatment of lung cancer. J Biomater Sci Polym Ed.

[CR262] Weissleder R, Stark DD, Engelstad BL, Bacon BR, Compton CC, White DL, Jacobs P, Lewis J (1989). Superparamagnetic iron oxide: pharmacokinetics and toxicity. AJR Am J Roentgenol.

[CR263] Wilhelm S, Tavares AJ, Dai Q, Ohta S, Audet J, Dvorak HF, Chan WC (2016). Analysis of nanoparticle delivery to tumours. Nat Rev Mater.

[CR264] Willowson KP (2019). Production of radionuclides for clinical nuclear medicine. Eur J Phys.

[CR265] Wong RM, Gilbert DA, Liu K, Louie AY (2012). Rapid size-controlled synthesis of dextran-coated, 64Cu-doped iron oxide nanoparticles. ACS Nano.

[CR266] Wu J (2021). The enhanced permeability and retention (EPR) effect: the significance of the concept and methods to enhance its application. J Pers Med.

[CR267] Wu J, Akaike T, Maeda H (1998). Modulation of enhanced vascular permeability in tumors by a bradykinin antagonist, a cyclooxygenase inhibitor, and a nitric oxide scavenger. Cancer Res.

[CR268] Wu J, Akaike T, Hayashida K, Okamoto T, Okuyama A, Maeda H (2001). Enhanced vascular permeability in solid tumor involving peroxynitrite and matrix metalloproteinases. Jpn J Cancer Res.

[CR269] Wu J, Akaike T, Hayashida K, Miyamoto Y, Nakagawa T, Miyakawa K, Müller-Esterl W, Maeda H (2002). Identification of bradykinin receptors in clinical cancer specimens and murine tumor tissues. Int J Cancer.

[CR270] Wu CY, Lin JJ, Chang WY, Hsieh CY, Wu CC, Chen HS, Hsu HJ, Yang AS, Hsu MH, Kuo WY (2019). Development of theranostic active-targeting boron-containing gold nanoparticles for boron neutron capture therapy (BNCT). Coll Surf B Biointerfaces..

[CR271] Xu Z, Wang Y, Han J, Xu Q, Ren J, Xu J, Wang Y, Chai Z (2018). Noninvasive multimodal imaging of osteosarcoma and lymph nodes using a 99mTc-labeled biomineralization nanoprobe. Anal Chem.

[CR272] Yang S, Gao H (2017). Nanoparticles for modulating tumor microenvironment to improve drug delivery and tumor therapy. Pharmacol Res.

[CR273] Yang Y, Shao Q, Deng R, Wang C, Teng X, Cheng K, Cheng Z, Huang L, Liu Z, Liu X (2012). In vitro and in vivo uncaging and bioluminescence imaging by using photocaged upconversion nanoparticles. Angew Chem Int Ed Engl.

[CR274] Yang K, Hu L, Ma X, Ye S, Cheng L, Shi X, Li C, Li Y, Liu Z (2012). Multimodal imaging guided photothermal therapy using functionalized graphene nanosheets anchored with magnetic nanoparticles. Adv Mater.

[CR275] Yang L, Sundaresan G, Sun M, Jose P, Hoffman D, McDonagh PR, Lamichhane N, Cutler CS, Perez JM, Zweit J (2013). Intrinsically radiolabeled multifunctional cerium oxide nanoparticles for in vivo studies. J Mater Chem B.

[CR276] Yang Y, Sun Y, Cao T, Peng J, Liu Y, Wu Y, Feng W, Zhang Y, Li F (2013). Hydrothermal synthesis of NaLuF4:153Sm, Yb, Tm nanoparticles and their application in dual-modality upconversion luminescence and SPECT bioimaging. Biomaterials.

[CR277] Yang Z, Tian R, Wu J, Fan Q, Yung BC, Niu G, Jacobson O, Wang Z, Liu G, Yu G (2017). Impact of semiconducting perylene diimide nanoparticle size on lymph node mapping and cancer imaging. ACS Nano.

[CR278] Yeong C-H, Cheng M, Ng K-H (2014). Therapeutic radionuclides in nuclear medicine: current and future prospects. J Zhejiang Univ Sci B.

[CR279] Yong K-T (2012). Quantum dots for biophotonics. Theranostics.

[CR280] Zavaleta CL, Hartman KB, Miao Z, James ML, Kempen P, Thakor AS, Nielsen CH, Sinclair R, Cheng Z, Gambhir SS (2011). Preclinical evaluation of raman nanoparticle biodistribution for their potential use in clinical endoscopy imaging. Small.

[CR281] Zeglis BM, Lewis JS (2011). A practical guide to the construction of radiometallated bioconjugates for positron emission tomography. Dalton Trans.

[CR282] Zeng J, Jia B, Qiao R, Wang C, Jing L, Wang F, Gao M (2014). In Situ 111In-doping for achieving biocompatible and non-leachable 111In-labeled Fe3O4 nanoparticles. Chem Commun (camb).

[CR283] Zhan Y, Ai F, Chen F, Valdovinos HF, Orbay H, Sui H, Liang J, Barnhart TE, Tian J, Cai W (2016). Intrinsically Zirconium-89 labeled Gd2O2S: Eu nanoprobes for in vivo positron emission tomography and gamma-ray-induced radioluminescence imaging. Small.

[CR284] Zhang Y, Jeon M, Rich LJ, Hong H, Geng J, Zhang Y, Shi S, Barnhart TE, Alexandridis P, Huizinga JD (2014). Non-invasive multimodal functional imaging of the intestine with frozen micellar naphthalocyanines. Nat Nanotechnol.

[CR285] Zhang Q, Han L, Jing H, Blom DA, Lin Y, Xin HL, Wang H (2016). Facet control of gold nanorods. ACS Nano.

[CR286] Zhang X, Yao M, Chen M, Li L, Dong C, Hou Y, Zhao H, Jia B, Wang F (2016). Hyaluronic acid-coated silver nanoparticles as a nanoplatform for in vivo imaging applications. ACS Appl Mater Interfaces.

[CR287] Zhao Y, Pang B, Luehmann H, Detering L, Yang X, Sultan D, Harpstrite S, Sharma V, Cutler CS, Xia Y (2016). Gold nanoparticles doped with (199) Au atoms and their use for targeted cancer imaging by SPECT. Adv Healthc Mater.

[CR288] Zhou J, Yu M, Sun Y, Zhang X, Zhu X, Wu Z, Wu D, Li F (2011). Fluorine-18-labeled Gd3+/Yb3+/Er3+ co-doped NaYF4 nanophosphors for multimodality PET/MR/UCL imaging. Biomaterials.

[CR289] Zimmermann RG (2013). Why are investors not interested in my radiotracer? The industrial and regulatory constraints in the development of radiopharmaceuticals. Nucl Med Biol.

[CR290] Zolata H, Afarideh H, Davani FA (2016). Triple therapy of HER2+ cancer using radiolabeled multifunctional iron oxide nanoparticles and alternating magnetic field. Cancer Biother Radiopharm.

